# Medication use trajectories in myasthenia gravis pregnancies: a population-based drug utilization study

**DOI:** 10.1007/s00415-026-14115-2

**Published:** 2026-09-07

**Authors:** Jenny Linnea Victoria Lindroos, Nils Erik Gilhus, Susanna Brauner, Marte-Helene Bjørk, Jana Midelfart-Hoff, Carolyn E. Cesta, Kari Furu, Jacqueline M. Cohen

**Affiliations:** 1 Department of Clinical Medicine, University of Bergen, Faculty of Medicine, Postbox 7804, 5020 Bergen, Norway; 2 Centre for Fertility and Health, Norwegian Institute of Public Health, Oslo, Norway; 3 Department of Neurology, Haukeland University Hospital, Bergen, Norway; 4 Department of Clinical Neuroscience, Karolinska Institute, Stockholm, Sweden; 5 Department of Neurology, Karolinska University Hospital, Stockholm, Sweden; 6 Faculty of Health Studies at VID Specialized University, Bergen, Norway; 7 Department of Medicine Solna, Centre for Pharmacoepidemiology, Karolinska Institute, Stockholm, Sweden; 8 Department of Chronic Diseases, Norwegian Institute of Public Health, Oslo, Norway

**Keywords:** Neuromuscular diseases, Reproductive health, Female, Immunosuppressive agents, Pyridostigmine, Prednisolone

## Abstract

**Objective:**

To examine medication use trajectories from 1 year before pregnancy to 6 months postpartum in a population-based birth cohort of patients with myasthenia gravis (MG).

**Methods:**

We used harmonized and pooled national register data from Norway and Sweden (1994–2023) to identify MG pregnancies. Longitudinal dispensing records of symptomatic MG medications, oral corticosteroids (OCS), and non-steroidal immunosuppressant therapy (NSIST) were extracted from prescription registers, which were individually linked to hospital records in the national patient registers and demographic and clinical data in the medical birth registers. Group-based multi-trajectory modeling (GBMTM) was used to identify pregnancy clusters and typical mono- or polytherapy medication use trajectories until delivery.

**Results:**

Among 339 MG pregnancies, 201 (59%) used some MG medication during pregnancy or 1 year before. Four distinct multi-drug trajectories were identified: ‘Intermittent users,’ ‘Persistent symptomatic-only users,’ ‘Immunosuppressant plus symptomatic users,’ and ‘Immunosuppressant-only users.’ Most symptomatic medication users remained on a stable medication use trajectory during pregnancy. Increased use of OCS was overall rare, but occurred in around 10% of “Intermittent users”. ‘Immunosuppressant-only users’ had stable OCS use throughout the study period and frequent non-MG hospital contacts, but rarely contacts due to MG. Although MG-related admissions were infrequent overall, they were consistently higher among ‘Immunosuppressant plus symptomatic users’. Further, ‘Immunosuppressant plus symptomatic users’ often discontinued NSIST during pregnancy.

**Interpretation:**

Most patients had no, low, or stable medication use throughout pre-pregnancy and pregnancy. Medication use trajectories discriminated MG patients with similar phenotypes and distinct healthcare utilization patterns. Such subgroups likely represent distinct disease courses in relation to pregnancy which could help in the development of individualized care pathways.

**Supplementary Information:**

The online version contains supplementary material available at https://doi.org/10.1007/s00415-026-14115-2.

## Introduction

Myasthenia gravis (MG) is an autoimmune, neuromuscular disease often requiring pharmacological treatment, also during pregnancy [18]. While unnecessary medications should be avoided to minimize fetal exposure, insufficient immunosuppression poses a risk to both mother and child [3, 4, 44].

Pharmacotherapy for MG includes both symptomatic and immunosuppressive treatments. Acetylcholine-esterase inhibitors (AChEI), such as pyridostigmine, are considered safe during pregnancy [19, 20]. The oral corticosteroid (OCS) prednisolone, which is the first-line immunosuppressant, is also considered safe at daily doses below 10 mg [32, 38, 40], whereas higher doses have been associated with preterm birth and long-term adverse child health outcomes [35, 40]. Among non-steroidal immunosuppressant therapies (NSIST), azathioprine is generally considered safe during pregnancy based on both observational studies and animal data, whereas cyclosporine and tacrolimus have shown toxicity in animal studies at higher doses [37]. Methotrexate, mycophenolate mofetil, and cyclophosphamide, on the contrary, are known teratogens and should not be used in women with reproductive potential [20, 32, 37]. Biologics, such as rituximab, are widely used in some countries, but not recommended in pregnancy [12, 27, 42, 46, 47]. Complement inhibitors and neonatal Fc-receptor antagonists are not universally reimbursed for MG patients in the Nordic countries and access is limited to very few patients [19]. Mild disease fluctuations can often be managed by adjusting peroral medication, but severe exacerbations require hospitalization, intravenous immunoglobulin (IVIG), plasma exchange (PLEX), or other rapidly acting treatments.

Treatment strategy is defined by disease severity, but also by side effects and concerns for the child. Studies have shown that many women with MG worry that their medication could harm their child, influencing reproductive decisions [2, 33]. Pregnancy is associated with MG worsening or relapse in around one-third of all MG pregnancies [43] and the disease course is unpredictable [5, 15]. Deterioration of MG during pregnancy may increase the risk of adverse pregnancy outcomes, including the risk of miscarriage, preterm birth, cesarean section, and transient neonatal myasthenia gravis [45]. Disentangling the effect of maternal disease from in utero medication exposure on fetal development is challenging and requires detailed understanding of actual treatment patterns in relation to sensitive fetal developmental windows and disease course [48].

Few studies have reported on MG medication use during pregnancy, and longitudinal analyses have been limited to preconception vs. pregnancy, early vs. late pregnancy, and during pregnancy vs. postpartum [14, 24]. Treatment patterns across more than two time periods at a time have only been described in a small number of patients [5, 15, 23, 24, 30]. Longitudinal clustering methods, such as the group-based multi-trajectory modeling (GBMTM), provide new opportunities to study such high-dimensional individual medication use data from multiple repeated measures, which allows us to estimate the total medication use across the whole course of pregnancy and typical medication use patterns, including treatment initiations, discontinuations, and switches; without a priori assumptions [31]. The model subgroups individuals with similar longitudinal medication use patterns, thereby identifying distinct medication use trajectories and potentially patients with unmet treatment needs [48].

Our aim was to describe MG medication use from 1 year before conception to 6 months postpartum in a population-based MG birth cohort, and to characterize pregnancies with similar MG medication use trajectories using demographic and clinical factors.

## Methods

### Data sources, study design, and setting

This register-based study utilized data from two population-based birth cohorts compiled from the medical birth registers (MBRs) of Norway 1999–2023 and Sweden 1994–2022, to which there is mandatory registration of all births from 22 completed gestational weeks. Data were harmonized in a common data model and pooled across countries with individual linkage on the mother’s unique national identity number [11]. The linkage included data from the prescribed drug registers (PDRs), going back to January 1, 2004 in Norway and July 1, 2005 in Sweden, and the national patient registers (NPRs), going back to 2008 in Norway and 1994 in Sweden (including specialist outpatient care since 2001). Data on maternal education level was obtained from Statistics Norway and Statistics Sweden. For the Swedish data, linkage was restricted to 5 years prior to and 3 years after each pregnancy.

All purchased prescribed medications are registered in the PDRs according to the Anatomical Therapeutic Chemical index (ATC) with the date of dispensing and the total dispensed amount [29]. NPRs cover all publicly funded inpatient and outpatient specialist care, which all residents in Norway and Sweden have access to through the universal healthcare systems. Less than 1% of outpatient MG care is delivered by private specialists, whereas the public sector delivers all obstetric and hospital in-patient care services for MG patients [16]. NPR includes the dates of hospital contacts, diagnoses coded by the International Classification of Diseases (ICD) 10th revision, or the 9th revision until 1996 in Sweden, and procedures coded by the Norwegian procedure coding system (NKPK) and the Swedish classification system for healthcare procedures (KVÅ), respectively [22, 41].

### Study population

The study population included all pregnancies ending in birth, i.e., live or stillbirth, if maternal MG was recorded before or during pregnancy and the birth date was after April 20, 2007 in Sweden, or October 21, 2009 in Norway. Thus, we ensured minimum 1 year of combined data coverage for prescription fills and healthcare data before the start of pregnancy, which was defined as the 1st day of the last menstrual period (LMP) calculated by subtracting the best clinical estimate of gestational age in days from the delivery date. The best clinical estimate of gestational age was in >90% of cases based on second trimester ultrasound measurement [8, 21]. No potentially eligible MG pregnancies had missing gestational age.

Maternal MG was identified from MBRs, NPRs, and PDRs using all available data until the date of delivery and predefined as (1) ≥ 1 MG diagnosis (ICD-10: G70.0 or ICD-9: 358A) on the birth notification from the index pregnancy or from any prior pregnancy (in MBR); (2) ≥ 2 MG diagnoses from specialist care ≥ 30 days apart (in NPR); (3) ≥ 2 prescription fills of pyridostigmine (ATC: N07AA02; in PDR); or (4) one diagnosis and one prescription fill of pyridostigmine. A validation study in Norway gave a positive predictive value of 99% for the MG diagnosis in MBR [23]. In a previous study, MG was identified with > 80% sensitivity by combining NPR and PDR in Sweden [17]. Only one diagnosis from specialist care only or only one prescription fill of pyridostigmine was not considered to be sufficient evidence of MG.

Since MG may manifest during pregnancy [6], in a secondary restricted definition we required maternal MG to be recognized before the start of pregnancy, thereby excluding cases that did not fulfill the above criteria before LMP, or based solely on an MBR diagnosis in the index pregnancy.

### Medications and medication use periods

We included all drugs that could be prescribed for MG and were dispensed at pharmacies during the study period (Table S1). Medications were classified as (1) symptomatic, including pyridostigmine, ambenonium chloride, amifampridine, and ephedrine; (2) OCS, including all oral corticosteroids such as prednisolone; or (3) NSIST, including azathioprine, cyclosporine, tacrolimus, voclosporin, cyclophosphamide, mycophenolate mofetil, methotrexate, and subcutaneous immunoglobulins.

Longitudinal medication use data was included from 1 year before pregnancy until 6 months postpartum (Table S2). We defined any use (≥ 1 prescription fill), total dispensed dose, and refill frequency for each medication and each medication class across nine time periods. These included four pre-pregnancy periods (lasting 91–92 days): 9–12 months, 6–9 months, 3–6 months, and 0–3 months before LMP; three pregnancy periods: first (91 days), second (max. 105 days), and third trimester (max. 110 days); and two postpartum periods (both 91 days): 0–3 months, and 3–6 months after delivery. The 3-month length of each period corresponds to the standard dispensed supply for chronically used medications in Norway and Sweden. Pregnancy periods were adapted to better align with standard trimester definitions [39] and varied depending on gestational length.

### Descriptive variables

We included obstetric and demographic background variables from MBR. To estimate the comorbidity burden, we defined a polypharmacy count as the number of distinct MG and non-MG medication purchases in PDR at the third ATC level (i.e., pharmacological subgroup) during pre-pregnancy or pregnancy, which included prescribed dietary supplements. Autoimmune comorbidity was defined from NPR as ≥ 1 diagnosis during pre-pregnancy or pregnancy of any of the 26 pre-selected autoimmune conditions regarded as associated with MG (Table S3) [17].

Hospital contacts included all inpatient admissions and outpatient visits to somatic specialist care, thus excluding psychiatric contacts. Hospital contacts with MG as the primary diagnosis were considered due to MG (MG admissions/MG visits), whereas non-MG admissions/non-MG visits had no MG code, neither as primary nor as secondary. For each contact category (MG, non-MG, and any admissions/visits), we counted the number of pregnancies with any number of such contacts, as well as the total number per person during pre-pregnancy, pregnancy (including the date of delivery), and postpartum, respectively.

### Statistical analysis

Using GBMTM [31], an unsupervised clustering method, we modeled medication use trajectories from 1 year before pregnancy until delivery among all pregnancies with any MG medication use during this period, referred to below as ‘Users’. This method can identify individual trajectories as growth curves from longitudinal data, so that the three medication classes (symptomatic medications, OCS, and NSIST) could depend on each other, thereby allowing polytherapy and switching between classes. We applied a logistic model where our binary medication use definitions functioned as dependent and the first seven time periods as independent variables (example code in Table S4).

The assumption of GBMTM is that the population consists of unobserved (latent) groups of individuals that have similar, distinct propensities to follow a certain trajectory. The model automatically assigned each pregnancy to one of a set number of latent groups so that the fit between individual and average multi-medication trajectories were maximized based on the posterior probability, i.e., each pregnancy’s likelihood of belonging to a group. The number of groups needs to be defined by the researcher based on clinical interpretability and a range of parameters describing model fit (posterior probability, odds of correct classification, and the Bayesian information criterion). We tested three to five groups. The five-group model consistently produced groups with < 10 pregnancies, which we had decided to avoid a priori due to personal data protection, clinical utility, and model reliability, whereas the four-group model produced more detailed information compared to only three groups. We arrived at the final model after testing several polynomial combinations up to the fourth polynomial ad hoc*,* using graphical output to guide our settings initially. For the final iterations, we prioritized good model fit. We report entropy for the final model as a summary measure of classification uncertainty, which takes a value from 0 to 1, where 1.0 indicates perfect classification, i.e., everyone clearly belongs to only one group. Results and model-fit statistics for alternative models are provided in Table S5.

Descriptive counts, averages, and proportions are reported separately by country, for ‘Users’ versus ‘Non-users’, and by trajectory group. For personal data protection we suppressed small cell counts < 5 and related total counts. Standardized differences, comparing each medication use trajectory group to ‘Non-users’, were calculated as Cohen’s h for proportions and Cohen’s d for means. Values < 0.2 were not considered meaningfully different [9]. Analyses were performed in Stata version 17.0/SE (StataCorp LLC). For the GBMTM, we used the *traj* package for Stata from https://www.andrew.cmu.edu/user/bjones/traj [26]. Figures were prepared in Stata and Microsoft Excel (Microsoft Corporation).

### Study registration

The study protocol was preregistered on the Open Science Framework (https://doi.org/10.17605/OSF.IO/TU7FY).

## Results

### Description of the cohort

Among a total of 2,531,740 births during the study period, we identified 339 MG pregnancies (108 pregnancies among 77 mothers in Norway and 231 pregnancies among 152 mothers in Sweden). These included < 5 multiple births, < 5 stillbirths, and 30 (8.8%) preterm births, of which 5 (1.5%) were born extremely early (22–28 gestational weeks). Maternal MG could be defined with certainty before pregnancy in 296 pregnancies of 200 mothers. In addition, 13 pregnancies had either one maternal MG diagnosis or one pyridostigmine fill, although not both, registered before LMP. In 27 pregnancies, the pre-pregnancy period overlapped with a previous pregnancy (mean interpregnancy interval 240 days, SD 83, range 37–349). Very long hospital stays (> 30 days) during the study period were rare (< 5 individuals).

The average maternal age at the time of delivery was 31.7 years (SD 4.7), and 151 (44.5%) pregnancies were the mother’s first (Table 1). Baseline characteristics were similar for Norway and Sweden (Table S6). On average, mothers purchased 6.3 (SD 4.6) different MG and non-MG medications or prescribed dietary supplements during pregnancy or 1 year before. After the “Nervous system” class, most prescribed drugs belonged to the “Anti‑infectives for systemic use” and “Respiratory system” classes.

**Table 1:** Clinical and demographic characteristics of all myasthenia gravis (MG) births in Norway (2010–2023) and Sweden (2007–2022), stratified by trajectory group

Characteristic, *n* (%)	All	‘Non-users'	‘Intermittent users’	‘Persistent symptomatic-only users’	‘Immunosuppressant plus symptomatic users’	‘Immunosuppressant-only users’
Total	339 (100)	138 (41)	71 (21)	67 (20)	35 (10)	28 (8)
Mother's age at the time of delivery, years^a^ (mean ± SD)	31.7 ± 4.7	31.6 ± 4.8	32.4 ± 4.0	31.5 ± 5.4	31.0 ± 4.0	32.0 ± 4.8
19–30	131 (39)	50 (36)	21 (30)	32 (48)	17 (49)	11 (39)
31–34	112 (33)	49 (36)	29 (41)	14 (21)	11 (31)	9 (32)
35–43	96 (28)	39 (28)	21 (30)	21 (31)	7 (20)	8 (29)
Number of previous births:						
0	151 (45)	55 (40)	36 (51)	32 (48)	16 (46)	12 (43)
1	138 (41)	61 (44)	25 (35)	26 (39)	15–18 (43﻿–51)	11 (39)
2 or more	47–50 (14﻿–15)	22 (16)	10 (14)	9 (13)	< 5 (3–11)	5 (18)
Previous stillbirth(s)	5 (2)	< 5 (1–3)	< 5 (1–6)	< 5 (1–6)	0 (0)	0 (0)
Year of delivery^b^ (range)	2007–2023	2007–2023	2007–2023	2007–2023	2008–2023	2008–2023
2007–2013	121 (36)	45 (33)	25 (35)	36 (54)	10 (29)	5 (18)
2014–2018	114 (34)	50 (36)	24 (34)	18 (27)	15 (43)	7 (25)
2019–2023	104 (31)	43 (31)	22 (31)	13 (19)	10 (29)	16 (57)
Source country = Norway	108 (32)	44 (32)	27 (38)	9 (13)	16 (46)	12 (43)
Mother born outside the Nordics^c^	83 (25)	25 (18)	22 (31)	17 (25)	13 (37)	6 (21)
Mother cohabiting	308 (91)	129 (94)	65 (92)	57 (85)	32 (91)	25 (89)
Mother's educational level:						
Bachelor’s, master’s, or higher	150 (44)	74 (54)	32 (45)	19 (28)	14 (40)	11 (39)
Secondary	144 (43)	52 (38)	31 (44)	38 (57)	12 (34)	11 (39)
Compulsory or less	36 (11)	11 (8)	6 (9)	7 (10)	6 (17)	6 (21)
Missing	9 (3)	< 5 (1–3)	< 5 (1–6)	< 5 (1–6)	< 5 (3–11)	0 (0)
Mother's BMI at first contact during pregnancy (mean ± SD)	25.3 ± 5.2	25.0 ± 5.4	25.4 ± 4.9	24.7 ± 5.0	27.3 ± 5.5	25.0 ± 5.0
BMI is missing	36 (11)	16 (12)	9 (13)	6 (9)	< 5 (3–11)	< 5 (4–14)
Non-smoking at the start of pregnancy^d^	297 (88)	129 (94)	57 (80)	58 (87)	28 (80)	25 (89)
Folic acid before pregnancy^e^	46–49 (43﻿–45)	24 (55)	9 (33)	5 (56)	< 5 (3–11)	7 (58)
Polypharmacy count^f^ (mean ± SD)	*6.3* ± *4.6*	*4.0* ± *3.5*	*6.7* ± *4.4*	*7.7* ± *4.3*	*8.5* ± *3.4*	*10.0* ± *6.6*
Chronic asthma	29 (9)	7 (5)	9 (13)	10 (15)	< 5 (3–11)	< 5 (4–14)
Diabetes	5 (2)	< 5 (1–3)	< 5 (1–6)	0 (0)	< 5 (3–11)	0 (0)
Gestational hypertension	8 (2)	< 5 (1–3)	< 5 (1–6)	< 5 (1–6)	< 5 (3–11)	0 (0)
Autoimmune comorbidity^g^	58 (17)	24 (17)	10 (14)	7 (10)	6 (17)	11 (39)
ART	23 (7)	7 (5)	6 (9)	< 5 (1–6)	< 5 (3–11)	6 (21)
Preterm birth (< 37 gw)	30 (9)	10 (7)	5–10 (7–14)	5–10 (7–15)	5 (14)	< 5 (4–14)

*n* number, *SD* standard deviation, *BMI* body mass index, *gw* gestational weeks, *ART* assisted reproductive technology, *ATC* Anatomical Therapeutic Classification, *MG* myasthenia gravis, *NPR* National Patient Register, *LMP* last menstrual period

^a^Mother’s age at the time of delivery was summarized from a continuous variable and categorized into tertiles

^b^Year of delivery was summarized from a continuous integer variable and categorized into tertiles. We included pregnancies with birth dates after October 21, 2009 and until December 31, 2023 in Norway and after April 20, 2007 and until December 31, 2022 in Sweden

^c^The Nordics include Denmark, Finland, Iceland, Norway, and Sweden

^d^We defined non-smoking as active information of not smoking. The other category included actively provided information about smoking, actively not wishing to provide information, and uninformed missing

^e^Was only available in data from Norway

^f^The polypharmacy count was defined as the number of distinct medications during pre-pregnancy or pregnancy at the third ATC level (pharmacological subgroup), including both MG and non-MG medications

^g^Autoimmune comorbidity was defined as at least 1 autoimmune condition other than MG among 26 predefined autoimmune conditions regarded to be associated with MG (Table S3), recorded in NPR before LMP

### Overall MG medication use

**Fig. 1:**
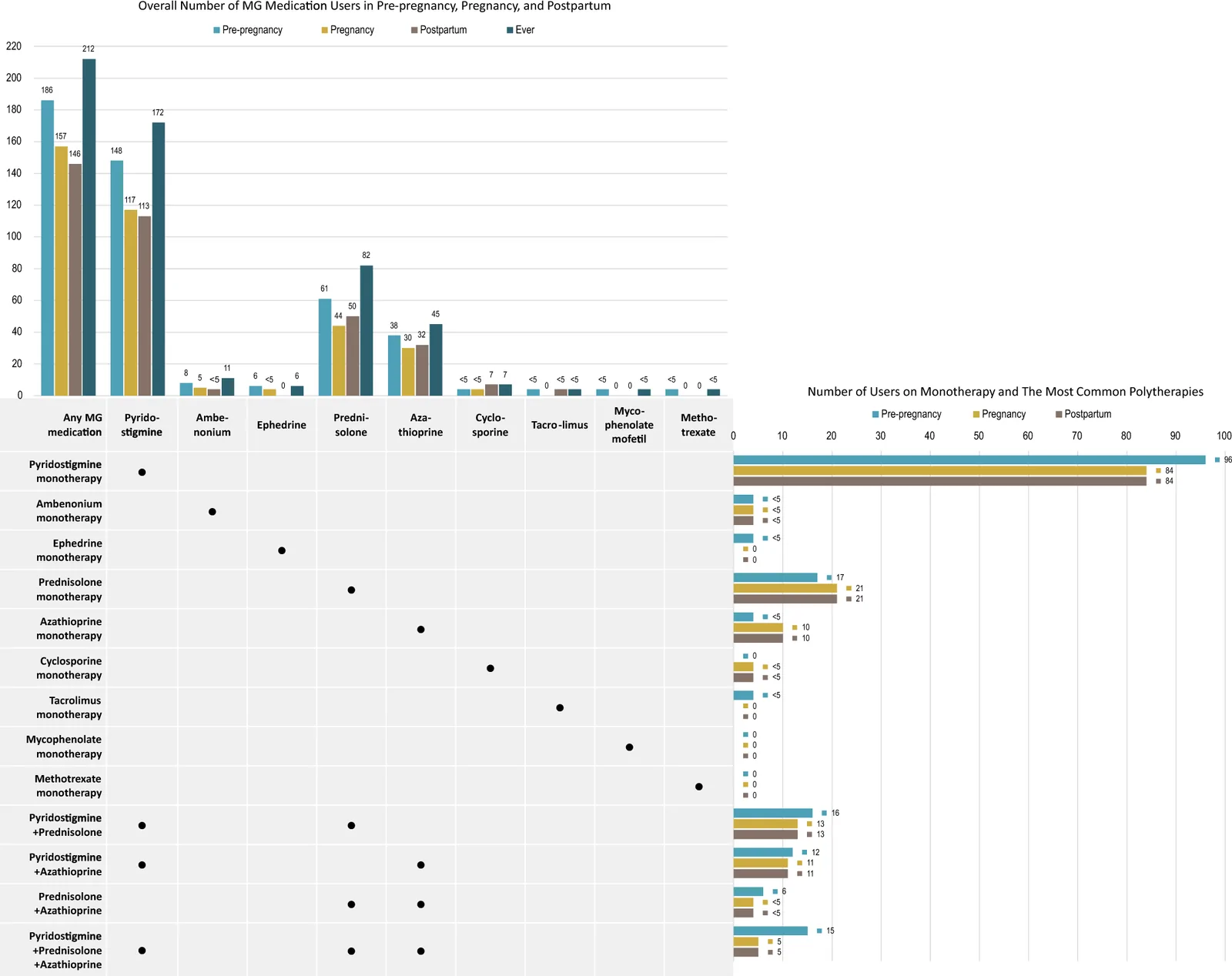
Number of users of any and each single MG drug overall (i.e., in mono- or polytherapy; vertical bars), and number of users of each single MG drug in monotherapy plus the most common MG drug combinations (horizontal bars); during the pre-pregnancy (1 year before LMP), pregnancy (LMP until 1 day before the date of delivery), and postpartum periods (the date of delivery until 6 months after), or ever (1 year before pregnancy to 6 months postpartum) among all MG births in Norway (2010–2023) and Sweden (2007–2022). Counts below five are suppressed and bar height is set to four (the top boundary). *MG* myasthenia gravis, *LMP* last menstrual period

Medication use decreased from pre-pregnancy to pregnancy for most MG drugs and combinations (Fig. 1). Teratogens (i.e., methotrexate, mycophenolate mofetil, and cyclophosphamide) were never dispensed later than 9–12 months pre-pregnancy. Tacrolimus was never refilled during pregnancy. All cyclosporine users (*n* = 7) were in Sweden (Table S7). Prednisolone was the only OCS used during the study period. We observed IVIG use in pre-pregnancy and pregnancy, rituximab use in pre-pregnancy and postpartum, and PLEX use in pre-pregnancy only (Table 2).

**Table 2:** MG therapies before delivery and up to 6 months postpartum among all MG pregnancies in the birth cohort, stratified by trajectory groups defined until delivery

MG therapy, *n* (%)	All	'Non-users'	‘Intermittent users’	‘Persistent symptomatic-only users’	‘Immunosuppressant plus symptomatic users’	‘Immunosuppressant-only users’
Total	339 (100)	138 (41)	71 (21)	67 (20)	35 (10)	28 (8)
Symptomatic MG medication^a^						
Pre-pregnancy or pregnancy, overall	169 (50)	0 (0)	61 (86)	67 (100)	35 (100)	6 (21)
Pre-pregnancy or pregnancy, monotherapy^b^	110 (32)	0 (0)	48 (68)	62 (93)	0 (0)	0 (0)
Postpartum:	115 (34)	10 (7)	19 (27)	57 (85)	28 (80)	< 5 (4–14)
0–3 months	86 (25)	8 (6)	10 (14)	46 (69)	21 (60)	< 5 (4–14)
3–6 months	97 (29)	6 (4)	16 (23)	47 (70)	28 (80)	0 (0)
OCS: ^c^						
Pre-pregnancy or pregnancy, overall	70 (21)	0 (0)	20 (28)	< 5 (1–6)	21 (60)	26 (93)
Pre-pregnancy or pregnancy, monotherapy^d^	18 (5)	0 (0)	8 (11)	0 (0)	0 (0)	10 (36)
Postpartum:	50 (15)	< 5 (1–3)	12 (17)	< 5 (1–6)	14 (40)	19 (68)
0–3 months	29 (9)	< 5 (1–3)	5 (7)	0 (0)	8 (23)	13 (46)
3–6 months	42 (12)	< 5 (1–3)	12 (17)	< 5 (1–6)	12 (34)	13 (46)
NSIST^e^						
Pre-pregnancy or pregnancy, overall	48 (14)	0 (0)	< 5 (1–6)	< 5 (1–6)	28 (80)	15 (54)
Pre-pregnancy or pregnancy, monotherapy^f^	< 5 (0–1)	0 (0)	< 5 (1–6)	0 (0)	0 (0)	< 5 (4–14)
Postpartum:	40 (12)	< 5 (1–3)	< 5 (1–6)	< 5 (1–6)	22 (63)	10 (36)
0–3 months	22 (7)	< 5 (1–3)	< 5 (1–6)	< 5 (1–6)	10 (29)	7 (25)
3–6 months	31 (9)	< 5 (1–3)	< 5 (1–6)	< 5 (1–6)	19 (54)	6 (21)
Rituximab (only for Norway from 2022)^d,g^						
Pre-pregnancy	2–8 (2–7)	< 5 (1–3)	< 5 (1–6)	0 (0)	0 (0)	0 (0)
Pregnancy	0 (0)	0 (0)	0 (0)	0 (0)	0 (0)	0 (0)
Postpartum	2–8 (2–7)	0 (0)	< 5 (1–6)	0 (0)	0 (0)	< 5 (4–14)
IVIG (only for Norway)^h^						
Pre-pregnancy	< 5 (0–1)	0 (0)	< 5 (1–6)	0 (0)	0 (0)	0 (0)
Pregnancy	< 5 (0–1)	0 (0)	< 5 (1–6)	0 (0)	0 (0)	0 (0)
Postpartum	0 (0)	0 (0)	0 (0)	0 (0)	0 (0)	0 (0)
PLEX^i^						
Pre-pregnancy	< 5 (0–1)	0 (0)	0 (0)	0 (0)	0 (0)	< 5 (4–14)
Pregnancy	0 (0)	0 (0)	0 (0)	0 (0)	0 (0)	0 (0)
Postpartum	0 (0)	0 (0)	0 (0)	0 (0)	0 (0)	0 (0)

*MG* myasthenia gravis, *n* number, *OCS* oral corticosteroids, *NSIST* non-steroidal immunosuppressive therapy

^a^Pyridostigmine, ambenonium chloride, or ephedrine

^b^No OCS or NSIST use during pre-pregnancy or pregnancy

^c^Prednisolone

^d^No symptomatic MG medication or NSIST use during pre-pregnancy or pregnancy

^e^Azathioprine, cyclosporine, tacrolimus, mycophenolate mofetil, or methotrexate

^f^No symptomatic MG medication or OCS use during pre-pregnancy or pregnancy

^g^Information on rituximab, given intravenously in hospital, was only reliably recorded year 2022–2023 and only available in the Norwegian dataset (NKPK: WBGM00*L01XC02 or WBGM00*L01FA01)

^h^Intravenous immunoglobulin treatment (NKPK: RPGM05) was only available in the Norwegian dataset

^i^Plasma exchange (NKPK: RPGD20 or RPGD25 in Norway; KVÅ: DR006 in Sweden)

During pre-pregnancy and pregnancy, there were 201 (59.3%) ‘Users’, i.e., individuals with at least one MG medication dispensing, whereas 138 (40.7%) were ‘Non-users’ and filled no MG prescriptions at all during pre-pregnancy or pregnancy. ‘Users’ were more often nulliparous (47.8% vs. 39.9%), born outside the Nordics (28.9% vs. 18.1%), more often assumedly smoking (83.6% vs. 93.5% were non-smoking), and seldom in the highest education category (37.8% vs. 53.6%; Table S8). ‘Users’ used on average 7.8 (SD 4.7) distinct prescribed drugs/dietary supplements during pre-pregnancy and pregnancy, whereas ‘Non-users’ used only 4.0 (SD 3.5).

### MG medication use trajectories before and during pregnancy

**Fig. 2:**
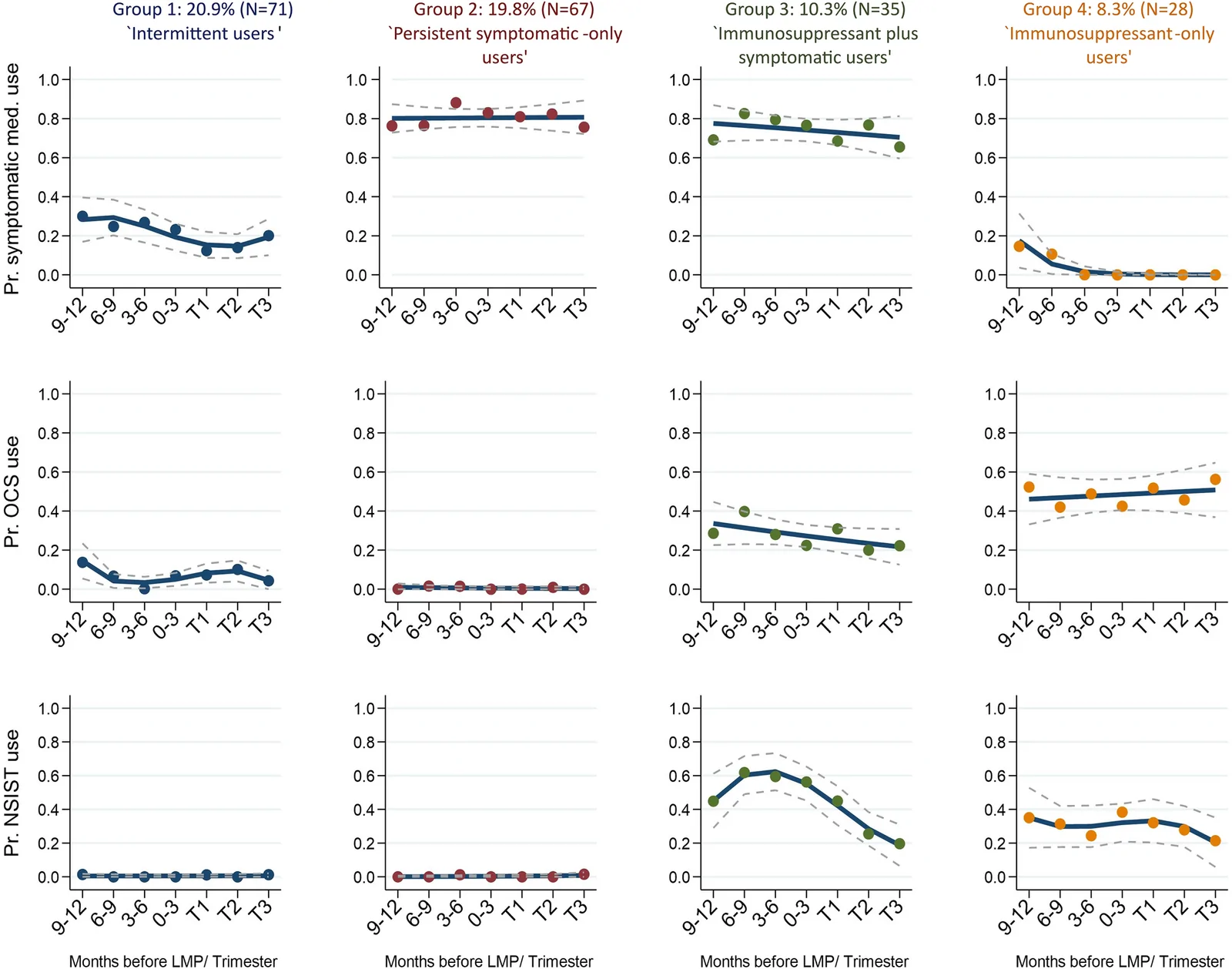
Multi-drug trajectory groups and longitudinal MG medication use patterns before and during pregnancy identified using group-based multi-trajectory modeling, including all MG pregnancies where the mother used any MG medication from 1 year before pregnancy to delivery (i.e., ‘Users’, *N* = 201). Group sizes were automatically derived from the model, here shown as percentages among the whole MG birth cohort (*N* = 339), which included ‘Non-users’. Dots illustrate the actual (observed) proportion of pregnancies filling at least one prescription of (1) any symptomatic MG medication, (2) any OCS, and/or (3) any NSIST within each drug use period. Lines illustrate the predicted probability of use across drug use periods, with 95% confidence intervals. *MG* myasthenia gravis, *N* number, *Pr*. probability, *OCS* oral corticosteroids, *NSIST* non-steroidal immunosuppressant therapy, *LMP* last menstrual period, *T1* trimester 1, *T2* trimester 2, *T3* trimester 3

Our four-group multi-trajectory model identified the following drug utilization patterns of MG medications: (‘Intermittent users’: 20.9%, *n* = 71; ‘Persistent symptomatic-only users’: 19.8%, *n* = 67; ‘Immunosuppressant plus symptomatic users’: 10.3%, *n* = 35; and ‘Immunosuppressant-only users’: 8.3%, *n* = 28; Fig. 2). Model entropy (0.92) and average posterior probabilities were very high (95.0–99.6%; Table S9). Persistence patterns of combination therapies and monotherapies are illustrated by alluvial diagrams in Fig S1. Within-group heterogeneity is illustrated by spaghetti plots in Fig. S2.

**Fig. 3:**
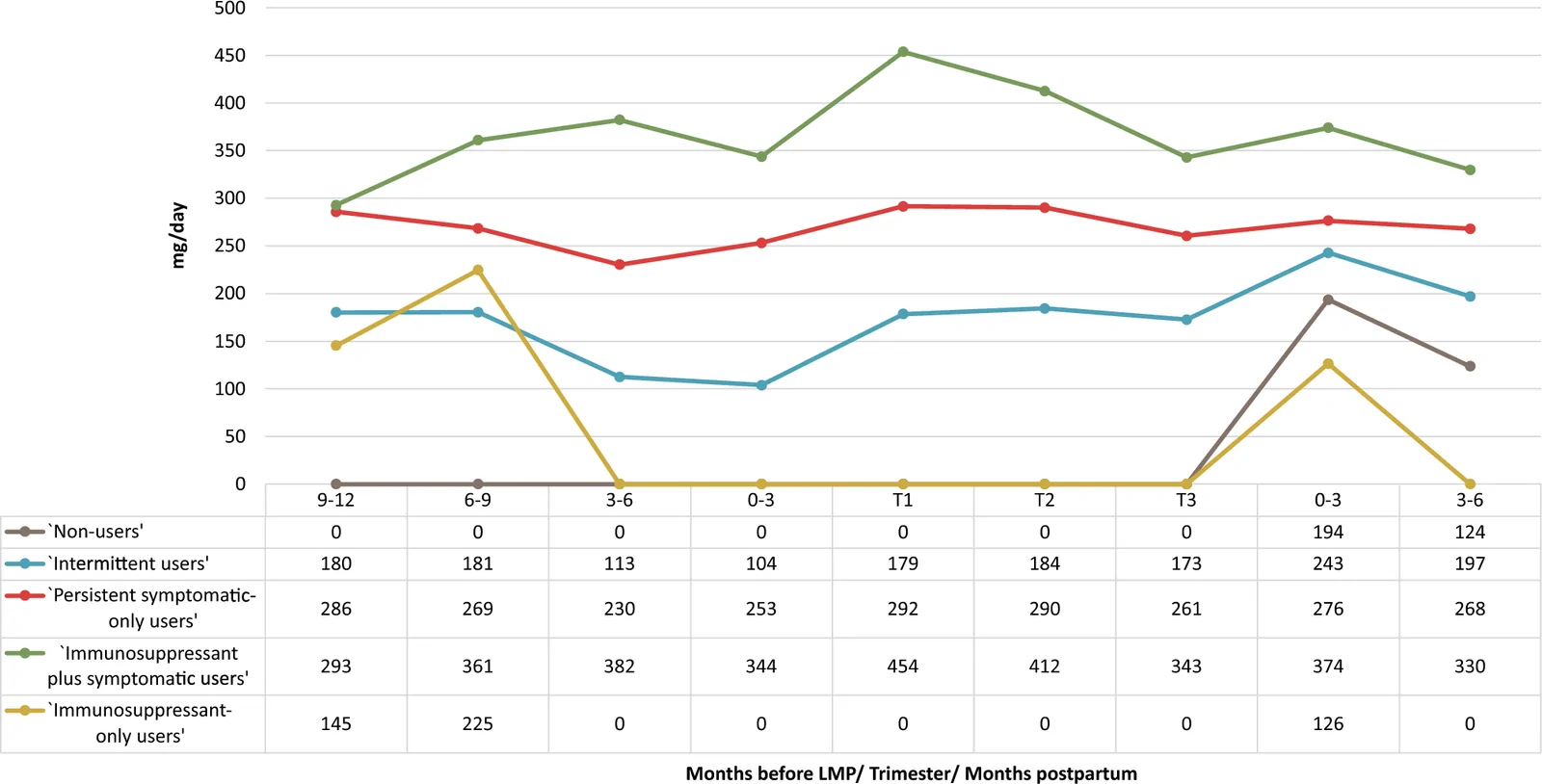
Average dispensed dose (mg/day) of pyridostigmine among users of pyridostigmine in each drug use period before, during, and after pregnancy, by trajectory group. The estimated dispensed daily dose per person was calculated as the total dispensed dose during the period and divided by 91 days, similar for all periods. Note that the actual length of the second trimester could vary between 64 and 105 days, and the third trimester could vary between 0 and 110 days

The two largest trajectory groups, ‘Intermittent users’ and ‘Persistent symptomatic-only users’, had no continuous immunosuppressant use and differed mainly in their longitudinal pattern of symptomatic medication use. In each pre-pregnancy and pregnancy period, 75–88% of ‘Persistent symptomatic-only users’ refilled symptomatic prescriptions (on average 2.0 times/period; Fig. S3). In contrast, only 21–28% of ‘Intermittent users’ filled symptomatic prescriptions during the pre-pregnancy period (on average 1.3 times/period), and only 13% during the second trimester. Despite this, 86% of ‘Intermittent users’, and all ‘Persistent symptomatic-only users’, were ever-users of symptomatic medications during pre-pregnancy or pregnancy (Table 2). The mean dispensed dose of pyridostigmine was significantly higher in ‘Persistent symptomatic-only users’ compared to ‘Intermittent users’ (269 vs. 159 mg/day during pre-pregnancy and pregnancy; Fig. 3). Some ‘Intermittent users’ had intermittent use of OCS, peaking at 10% in the second trimester. Less than five ‘Persistent symptomatic-only users’ used OCS temporarily.

‘Immunosuppressant plus symptomatic users’ had persistent use of symptomatic medications, whereas ‘Immunosuppressant-only users’ either used no symptomatic medication (79%) or had their last symptomatic medication dispensing before pregnancy (21%; Fig. 2). During pre-pregnancy and pregnancy combined, 60% of ‘Immunosuppressant plus symptomatic users’ and 93% of ‘Immunosuppressant-only users’ used OCS during the study period, and 80% and 54% used NSIST, respectively. Stand-alone immunotherapy with either OCS or NSIST (with or without symptomatic medications) accounted for 20% and 40%, respectively, among ‘Immunosuppressant plus symptomatic users’ and for 46% and < 14%, respectively, among ‘Immunosuppressant-only users’. The remaining individuals used both OCS and NSIST during pre-pregnancy or pregnancy. NSIST use decreased during pregnancy in both groups, most prominently among ‘Immunosuppressant plus symptomatic users’ (from ~ 60% filling in pre-pregnancy to 20% in the third trimester). ‘Immunosuppressant plus symptomatic users’ also had decreasing use of OCS toward term, while OCS use increased slightly among ‘Immunosuppressant-only users’.

‘Immunosuppressant plus symptomatic users’ consistently purchased the highest doses of pyridostigmine, particularly in the first trimester (450 mg/day on average; Fig. 3). ‘Immunosuppressant-only users’ had a very stable dose pattern of prednisolone around 8 mg/day throughout pre-pregnancy and pregnancy (Fig. S4), including a very stable refill frequency (on average 1.3 fills/period; Fig. S4). ‘Immunosuppressant plus symptomatic users’, on the other hand, had a more varying dose pattern and consistently purchased higher amounts of prednisolone (16 mg/day on average; Fig. S5). The dispensed doses of azathioprine were similar for these two groups during pre-pregnancy, but in the second and third trimesters ‘Immunosuppressant plus symptomatic users’ tended to have higher amounts than ‘Immunosuppressant-only users’ (Fig. S6).

In the sub-analysis of pregnancies where maternal MG was confirmed before the start of pregnancy, multi-trajectory patterns were largely preserved, but with a sharper decline of symptomatic medication use from pre-pregnancy to pregnancy among ‘Intermittent users’, which apart from ‘Non-users’ (*n* = 110 vs. 138) were most affected by this restriction (*n* = 60 vs. 71; Fig. S7; Table S10).

### Trajectory group characteristics

**Fig. 4:**
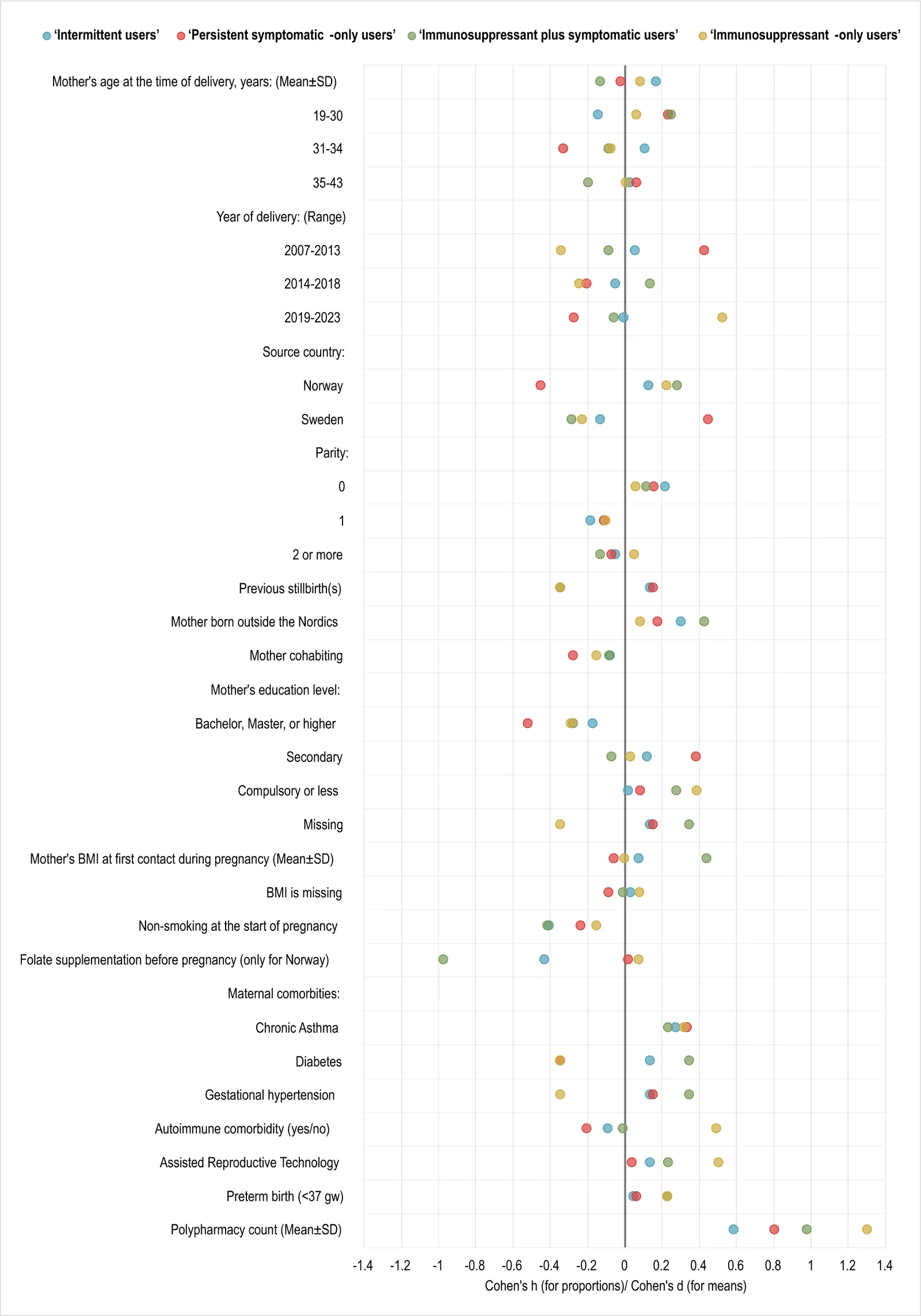
Standardized differences of clinical and demographic characteristics for each medication use trajectory group versus ‘Non-users’

Maternal age and parity were similar across groups, but ‘Persistent symptomatic-only users’ and ‘Immunosuppressant plus symptomatic users’, compared to ‘Non-users’, were more often 30 years or younger, whereas’Intermittent users’ tended to be older but more often nulliparous (Table 1, Fig. 4). ‘Persistent symptomatic-only users’, ‘Immunosuppressant plus symptomatic users’, and ‘Immunosuppressant-only users’ had generally achieved a lower level of education than ‘Non-users’. ‘Persistent symptomatic-only users’ were mostly from the earliest part of the study period (54%) and from Sweden (87%), whereas ‘Immunosuppressant-only users’ were often from Norway (43%) and the latest one-third of the study period (57%). Also ‘Immunosuppressant plus symptomatic users’ were often from Norway (46%). Both ‘Intermittent users’ and ‘Immunosuppressant plus symptomatic users’ were more often born outside the Nordic countries compared to ‘Non-users’. ‘Immunosuppressant plus symptomatic users’ had on average the highest BMI (27.3 ± 5.5) and had more often diabetes and gestational hypertension compared with ‘Non-users’. ‘Immunosuppressant plus symptomatic users’, ‘Intermittent users’, and ‘Persistent symptomatic-only users’ were more often smokers or had not given information on smoking. ‘Immunosuppressant plus symptomatic users’ and ‘Intermittent users’ had also less often used folic acid supplementation before pregnancy. ‘Immunosuppressant-only users’ had more often conceived through assisted reproductive technology (21%) and a high proportion of autoimmune comorbidity (39%). They also had the highest polypharmacy count, followed by ‘Immunosuppressant plus symptomatic users’, ‘Persistent symptomatic-only users’, ‘Intermittent users’, and ‘Non-users’.

### Hospital contacts by trajectory groups

**Fig. 5:**
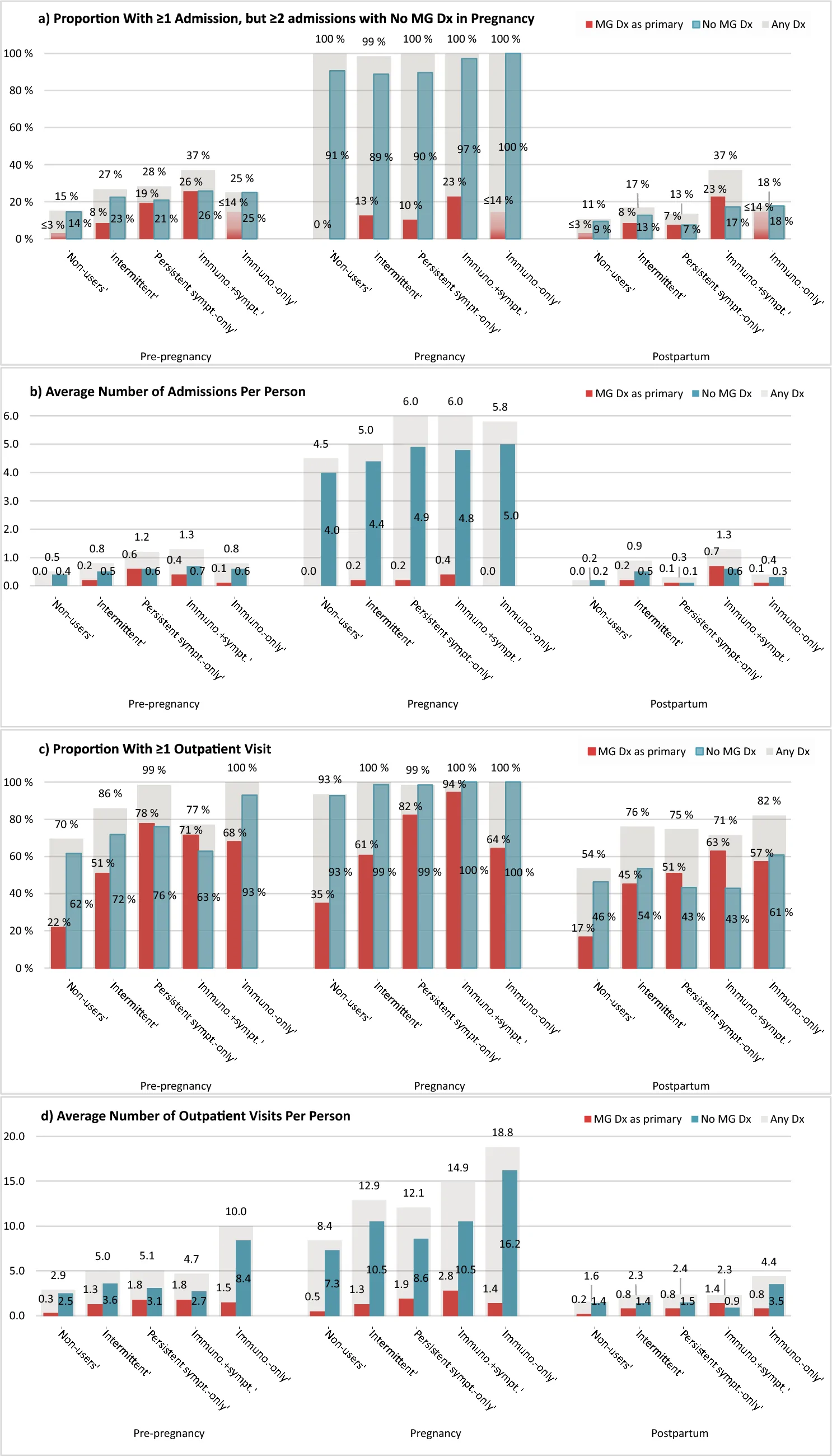
All somatic hospital contacts during the pre-pregnancy, pregnancy, and postpartum periods among all MG pregnancies in the birth cohort, stratified by trajectory group, by type of contact (inpatient [panel **a**, **b**]/outpatient [panel c and d]), and by Dx (MG [in red]/non-MG [in blue]﻿/any [in gray]). **a** Proportion of individuals in each trajectory group with any number of MG admissions/non-MG admission/any admissions during pre-pregnancy and postpartum or any number of MG admissions/any admissions during pregnancy, *but at least two non-MG admissions during pregnancy* (all participants had at least one non-MG admission during pregnancy, which included admission for delivery) **b** Average (mean) number of MG/non-MG/any admissions per person in each trajectory group. *During pregnancy, the average number above one is given for non-MG* **c** Proportion of individuals in each trajectory group with any number of MG/non-MG/any visits **d** Average (mean) number of MG/non-MG/any visits per person in each trajectory group. Hospital contacts due to MG had MG Dx (ICD-10: G70.0) in the primary position. Non-MG contacts had no MG Dx, neither as primary nor as secondary. Any Dx included all inpatient admissions/outpatient visits to somatic specialist care for any cause, including contacts with MG Dx in primary or secondary positions. The pre-pregnancy period included 365 days before LMP, pregnancy included the date of LMP to the date of delivery, and the postpartum period included 182 days after the date of delivery. Percentages of counts below 5 are suppressed. Descriptive trajectory group names are shortened from: ‘Non-users’, ‘Intermittent users’, ‘Persistent symptomatic-only users’, ‘Immuno-suppressant plus symptomatic users’, ‘Immuno-suppressant-only users’. *MG* myasthenia gravis, *Dx* diagnosis, *MP* last menstrual period

Among all individuals, 79 (23.3%) had at least one inpatient hospital admission in the pre-pregnancy period, of which 30 (8.8%) were due to MG. Admissions and outpatient visits for MG were most common among ‘Immunosuppressant plus symptomatic users’ (26% had ≥ 1 MG admission and 71% had ≥ 1 MG visit) and ‘Persistent symptomatic-only users’ (19% had ≥ 1 MG admissions and 78% had ≥ 1 MG visits; Fig. 5). In contrast, ‘Immunosuppressant plus symptomatic users’ (23%), ‘Non-users’ (30%), and ‘Intermittent users’ (14%) had the highest proportions of patients with no outpatient visit for any diagnosis in the pre-pregnancy period.

During pregnancy, virtually everyone had at least one admission to hospital (including for delivery) and 91.4% (*n* = 310) had more than one non-MG admission. MG admissions occurred most frequently among ‘Immunosuppressant plus symptomatic users’ (23%), never among ‘Non-users’, and rarely among ‘Immunosuppressant-only users’ (*n* < 5). In contrast, ‘Immunosuppressant-only users’ had many non-MG contacts, as in the pre-pregnancy period.

In the postpartum period, 54 (15.9%) women were readmitted to hospital, of which 23 (6.8%) due to MG. Similar to the pre-pregnancy and pregnancy periods, ‘Immunosuppressant plus symptomatic users’ had the highest proportion of patients with any number of MG admissions (23%) and any number of admissions overall (37%), while ‘Immunosuppressant-only users’ had high numbers of non-MG contacts, but rarely MG contacts.

### Medication use postpartum by trajectory groups

NSIST use increased among ‘Immunosuppressant plus symptomatic users’ during the first 3 months after delivery. At 6 months postpartum, NSIST use was back to pre-pregnancy levels (Table 2). ‘Immunosuppressant-only users’, on the other hand, remained on their reduced NSIST trajectory postpartum.

The number of OCS users increased postpartum among ‘Immunosuppressant plus symptomatic users’, ‘Intermittent users’, and ‘Non-users’, who also sometimes received dispensed doses exceeding 15 mg/day (Fig. S3), whereas ‘Immunosuppressant-only users’ continued their stable use and dose trajectory postpartum.

(Re-)initiation of symptomatic medications occurred in 10 (7%) ‘Non-users’ and in < 5 (4–14%) ‘Immunosuppressant-only users’.

## Discussion

In this large population-based birth cohort across two Nordic countries, we identified four distinct trajectories of MG medication use before and during pregnancy. Pregnancies within trajectory groups often shared clinical and demographic characteristics, including hospital contact patterns, signifying underlying similar phenotypes. Despite having only medication use data to discriminate groups, our data suggest that we have identified groups that are closely related to the standardized MG disease status classes as defined by the Myasthenia Gravis Foundation of America Post-intervention Status classification (MGFA-PIS) [25]. ‘Non-users’ likely signify complete stable remission or minimal manifestation type 0 (MM-0). ‘Immunosuppressant-only users’ may consist of individuals in pharmacologic remission, MM-1, or of patients with more active MG but with contraindications to or side effects from symptomatic medications. ‘Intermittent users’ may signify MM-2 before pregnancy, although some probably had new-onset MG and 10–15% had intermittent use of OCS and/or MG admissions as signs of MG worsening during pregnancy. ‘Persistent symptomatic-only users’ had evidence of stable, symptomatic MG in pregnancy and postpartum, whereas ‘Immunosuppressant plus symptomatic users’ either had MM-3 or active MG.

Most patients had low, stable, or reduced medication use in pregnancy and postpartum compared to pre-pregnancy. Most hospital contacts due to MG occurred in the outpatient setting and non-MG admissions dominated during pregnancy. Although ‘Immunosuppressant plus symptomatic users’ showed a reduction in the number of medication users during pregnancy, we observed an increase in dispensed doses of pyridostigmine and prednisolone over time, which may indicate worsening in some patients within this group. At the same time, this group had the highest number of hospital admissions due to MG during pregnancy and postpartum. A previous study found that MG worsening was more prevalent among individuals who discontinued immunosuppression during pregnancy [3]. The clear reduction of NSIST prescription fills after conception probably signifies an attempt to avoid fetal NSIST exposure, although we cannot exclude discontinuation due to MG improvement, side effects, or pregnancy-related concerns such as emesis. Interestingly, very few patients in the ‘Immunosuppressant plus symptomatic users’ group used folic acid supplements before pregnancy (*n* < 5), and many had no outpatient contact in the hospital at all during pre-pregnancy (23%), although this group scored high on all other hospital contact categories. This indicates that pregnancy probably was unplanned or at least not preceded by pre-pregnancy counseling with a specialist. Our findings emphasize the importance of pregnancy planning in coordination with neurology services, especially for patients needing immunosuppressive therapy [4].

A large group of patients, 41%, used no MG medications from 1 year before pregnancy to delivery. Similar findings have recently been reported from Poland (44%) [1], while the proportion of non-users was lower in a study from Germany (29%) [14], but higher in a study from the USA (61%) [24]. It is possible that pre-pregnancy use of new therapies, such as FcRn-, complement-, or B-cell inhibitors, which are currently recommended to be discontinued before pregnancy, explains the higher proportion of non-users in the US population. ‘Non-users’ had almost no MG admissions in pregnancy or postpartum and rarely initiated MG treatments postpartum. ‘Intermittent users’, the second largest group, also had low use of specialized healthcare services overall, second only to ‘Non-users’, with whom they shared most characteristics such as high education. Although the lack of NSIST use suggests that ‘Intermittent users’ had low baseline disease severity, the relatively high proportion of MG admission in pregnancy (13%) and postpartum (8%) and the intermittent high-dose OCS use indicate that a subset of ‘Intermittent users’ had MG worsening. Eleven individuals in this group, amongst 30 individuals in total, had evidence of new-onset MG during pregnancy. When these were excluded, there was a clear and sustained decline of symptomatic medication use. This suggests that those with only low use of pyridostigmine and/or occasional prednisolone in pre-pregnancy often remain stable or improve during pregnancy.

Although ‘Persistent symptomatic-only users’ rarely used immunosuppressant medications, they had high use of symptomatic medications throughout the whole study period, indicating symptomatic, albeit likely stable, disease. This was supported by a relatively high number of MG contacts in pre-pregnancy, but low numbers in pregnancy and postpartum. The strong adherence to symptomatic medications during pregnancy, which was typical, although not unique for this group, indicates that such medications were perceived beneficial and safe, in line with previous expert opinions [32, 37]. Since this group included predominantly Swedish pregnancies where rituximab use has become increasingly common, some patients had probably received rituximab treatment which we were unable to detect, as infusion drugs are not captured in national health registries. Immunosuppressant use was overall lower in Sweden compared to Norway, although cyclosporine and tacrolimus were more common, which likely reflects differences in treatment practice. 

Only 21% of ‘Immunosuppressant-only users’ used symptomatic medications in the pre-pregnancy period, but no-one refilled in the last 6 months before pregnancy or during pregnancy. It is possible that they to a large extent relied on OCS to treat MG symptoms, as this group exhibited a stable prednisolone trajectory with an average daily dose below 10 mg. Possible explanations for reducing symptomatic medications include good disease control, cholinergic side-effects, and a wish to avoid fetal exposure, although pyridostigmine is recommended always when perceived beneficial for symptom control as it is considered safe during pregnancy [19, 20]. This group rarely increased their medication use and they were very rarely admitted to hospital due to MG, indicating a low MG burden. However, they had frequent use of non-MG specialist services. Pregnancy-related complications associated with prednisolone use may partly explain their high healthcare needs. OCS use has been associated with hypertension, hyperglycemia, weight gain, infections, intrauterine growth restriction, preeclampsia, and preterm birth [35, 40]. High healthcare utilization could also be related to autoimmune comorbidity or fertility treatment, which was prevalent in this group.

Surprisingly, the use of NSIST, especially azathioprine, increased during the last 9 months before pregnancy among ‘Immunosuppressant plus symptomatic users’. In contrast, we found that teratogenic medications were always discontinued prior to pregnancy, although very few (*n* < 5) patients in our cohort used teratogens. In comparison to similar patient populations in the USA, this number is low [24, 36]. Teratogens should be completely avoided in females with reproductive potential [32]. The increasing use of azathioprine, which in part could have been a switch from teratogens or rituximab, indicates an attempt to optimize medications and MG status in preparation for pregnancy. Alternatively, the chance of conception may have naturally increased in a period of improved disease stability. Immunosuppressant treatment could also directly reduce the risk of autoantibody transmission from mother to child, including the risk of neonatal myasthenia gravis, independent of symptom reduction [5, 28]. As some patients had a previous pregnancy overlapping with the pre-pregnancy period, the upward-sloping NSIST trajectory may also represent a return to baseline.

### Strengths and limitations

Strengths of this study include a large, population-based MG cohort with prospectively recorded data. Demographics and MG care are similar in Norway and Sweden, and pooling data across the two countries increased our cohort size. Prescription register data of medications for chronic diseases, such as MG, correlate well with actual use outside and during pregnancy [7, 34]. We defined use in each period by the act of filling a prescription rather than supply into the period, since this represents stronger evidence of use, especially around pregnancy where adherence may drop [10]. Our longitudinal model followed individuals with several repeated measures, allowing us to make stronger inferences of actual use compared to more traditional methods that rely on predefined categories and a priori assumptions. In the presence of irregular medication purchase patterns, such as stockpiling, this approach is more flexible and less prone to misclassification compared to such arbitrary classification rules [48]. Group-based trajectory modeling has also been shown superior in evaluating adherence compared to k-means clustering and to the method “proportion of days covered” [13]. By employing the multi-drug option, we were able to evaluate mono- and polytherapy patterns simultaneously.

A limitation of our study is that we lacked information about infusion medications administered in hospitals in Sweden, and in Norway before 2022, most importantly rituximab. Rituximab is not recommended during the last 3 months before pregnancy [37], but its long-lasting immunosuppressive effect could still have influenced the MG course during pregnancy and consequently the need for other MG therapies. Unrecorded rituximab use may be one of the explanations for the high number of ‘Persistent symptomatic-only users’ (20% of the cohort) and the relatively low number of immunosuppressant users overall (24% OCS and 16% NSIST ever-users during the study period), especially in Sweden (19% OCS and 12% NSIST ever-users during the study period). Another limitation is that spontaneous and induced abortions could not be included since they are incompletely captured by the birth registers. Individuals with severe MG or medications that are harmful to the fetus may be more likely to experience abortion [15, 28, 45]. Such selection bias could have led to underestimation of medication use, especially teratogens. Our findings are thus best generalizable to MG pregnancies progressing to birth. Mothers with less severe MG may have been overrepresented if they contributed more pregnancies. Parity tended higher among ‘Non-users’, but differences were negligible. We did not have access to detailed clinical and biomarker data, such as MG severity scales, antibody and thymectomy status, and disease subgroup. Finally, we did not include data on malformations and perinatal complications in relation to trajectory groups as such associations would have required a different study design.

## Conclusion

In this register-based study, we described population-based medication use trajectories, identifying clinically distinct groups of MG pregnancies. Trajectory groups that were differentiated based on longitudinal data of dispensed MG medications have distinct hospital contact patterns, correspond to the MGFA-PIS classification, and may serve as proxies for disease severity. Women with MG who entered pregnancy using both immunosuppressant and symptomatic therapy often had fewer NSIST dispensings during pregnancy, but this group also had the highest number of MG hospital admissions. Many of them did not have any contact with specialist care during the last year before pregnancy. This emphasizes the importance of individualized management of MG and preemptive discussions about reproductive plans when treating women with MG of childbearing age.

## Supplementary Information

Below is the link to the electronic supplementary material.

Supplementary file1 (DOCX 1379 KB)

Supplementary file2 (DOCX 30 KB)

## Data Availability

Data are not publicly available due to privacy restrictions. Access to health register data may be applied from the respective register holders in each country.
