# Supplementary material for: Medication use trajectories in myasthenia gravis pregnancies: a population-based drug utilization study
Source: J Neurol. 2026 Sep 7;273(10):572. doi: 10.1007/s00415-026-14115-2 (PMC13550055; doi:10.1007/s00415-026-14115-2)
Supplement: Supplementary file 1 [file 415_2026_14115_MOESM1_ESM.docx]

Manuscript Title:

**Medication Use Trajectories in Myasthenia Gravis Pregnancies, a Population-Based Drug Utilization Study**

Short Title:

**Medication Trajectories in MG Pregnancies**

Journal:

**Journal of Neurology**

Authors:

**MD Jenny L. V. Lindroos^1,2,3^**

**MD, Prof. Nils Erik Gilhus^1,3^**

**MD, PhD Susanna Brauner^4,5^**

**MD, Prof. Marte-Helene Bjørk^1,3^**

**MD, Prof. Jana Midelfart-Hoff^5^**

**PhD Carolyn E. Cesta^7^**

**PhD Kari Furu^2,8^**

**PhD Jacqueline M. Cohen^2,8^**

Author Affiliations:

¹ Department of Clinical Medicine, University of Bergen, Bergen, Norway

² Centre for Fertility and Health, Norwegian Institute of Public Health, Oslo, Norway

³ Department of Neurology, Haukeland University Hospital, Bergen, Norway

⁴ Department of Clinical Neuroscience, Karolinska Institute, Stockholm, Sweden

⁵ Department of Neurology, Karolinska University Hospital, Stockholm, Sweden

^6^ Faculty of Health Studies at VID Specialized University, Bergen, Norway

^7^ Centre for Pharmacoepidemiology, Department of Medicine Solna, Karolinska Institute, Stockholm, Sweden

^8^ Department of Chronic Diseases, Norwegian Institute of Public Health, Oslo, Norway

Correspondence to:

Jenny Linnea Victoria Lindroos

Department of Clinical Medicine, University of Bergen, Faculty of Medicine

Postbox 7804, NO-5020 Bergen, Norway

Tel. +47 97 59 15 01, E-mail: [jenny.lindroos@uib.no](mailto:jenny.lindroos@uib.no)

Table of Contents

[Table S1. MG Medications Dispensed at Pharmacies and Definitions	4](#_Toc238629660)

[Table S2. Medication Use Periods and Definitions	5](#_Toc238629661)

[Table S3. Autoimmune Diseases Included in the Autoimmune Comorbidity Score, based on Fang et.al 2015 (doi:10.1111/joim.12310)	6](#_Toc238629662)

[Table S4. Code to Set Up and Run the Trajectory Model in Stata	9](#_Toc238629663)

[Table S5. Examples of Alternative Models and Model Fit Statistics	10](#_Toc238629664)

[Table S6. Clinical and Demographic Background Characteristics for the MG Pregnancy Cohort, Pooled and By Country	11](#_Toc238629665)

[Table S7. Proportion Medication Users of Each Medication and Medication Class in Each Drug Use Period, Pooled and By Country	14](#_Toc238629666)

[Table S8. Clinical and Demographic Background Characteristics for Cohort, and Separately by Pregnancies with Any MG Medication Use During Pre-pregnancy and Pregnancy (‘Users’) vs. No MG Medication Use (‘Non-users’)	17](#_Toc238629667)

[Table S9. Polynomial Settings, Model Fit Statistics, and GBMTM Results for the Final Main Model	20](#_Toc238629668)

[Table S10. Polynomial Settings, Model Fit Statistics, and GBMTM Results for the Restricted Cohort, i.e. MG Defined Until LMP	21](#_Toc238629669)

[Figure S1. Alluvial diagrams illustrating persistence patterns of combination-therapies and monotherapy across pre-pregnancy, pregnancy and postpartum, by trajectory groups.	22](#_Toc238629670)

[Figure S2. Spaghetti plots of individual predicted probabilities.	24](#_Toc238629671)

[Figure S3. Mean number of prescription fills of **pyridostigmine** among pyridostigmine users in each drug use period, by trajectory groups.	25](#_Toc238629672)

[Figure S4. Mean number of prescription fills of prednisolone among prednisolone users in each drug use period, by trajectory groups.	27](#_Toc238629673)

[Figure S5. Mean daily dose (mg/day) of prednisolone as the total dispensed dose during period averaged over 91 days among users of prednisolone in each period, by trajectory groups.	29](#_Toc238629674)

[Figure S6. Mean daily dose (mg/day) of azathioprine as the total dispensed dose during period averaged over 91 days among users of azathioprine in each period, by trajectory groups	31](#_Toc238629675)

[Figure S7. Multi-drug trajectory patterns before and during pregnancy, in the restricted birth cohort where myasthenia gravis was recognized before the start of pregnancy.	32](#_Toc238629676)

## Table S1. MG Medications Dispensed at Pharmacies and Definitions

| **Generic name** | **Short code** | **ATC-code** | **Comment** |  |
| --- | --- | --- | --- | --- |
| Symptomatic medications (AChEI or other): | | | | |
| Pyridostigmine | MEST | N07AA02 |  |  |
| Ambenonium chloride | MYT | N07AA30 |  |  |
| Amifampridine | DAP | N07XX05 | No prescription fills in cohort |  |
| Ephedrine | EPH | R03CA02 |  |  |
| OCS: | | | | |
| Prednisolone | PRED | H02AB06 |  |  |
| Any other oral corticosteroid (prednisone, betamethasone, dexamethasone, fluocortolone, methylprednisolone, paramethasone, hydrocortisone, cortisone, prednylidene, deflazacort, cloprednol, meprednisone, cortivazol, vamorolone) | CORT | H02AB, excluding: H02AB06 (prednisolone), H02AB08 (triamcinolone, no oral preparation in Norway or Sweden), H02AB12 (rimexolone, only intra-articular) | No prescription fills in cohort |  |
| NSIST: | | | | |
| Azathioprine | AZA | L04AX01 |  |  |
| Cyclosporine A | CSPA | L04AD01 |  |  |
| Tacrolimus | TAC | L04AD02 |  |  |
| Voclosporin | VOC | L04AD03 | No prescription fills in cohort |  |
| Mycophenolate mofetil | MMF | L04AA06 |  |  |
| Methotrexate | MTX | L01BA01 |  |  |
| Cyclophosphamide | CPH | L01AA01 | No prescription fills in cohort |  |
| Subcutaneous immunoglobulin | SCIG | J06BA01 | No prescription fills in cohort |  |

Abbreviations: MG = Myasthenia Gravis; ATC = Anatomical Therapeutic Chemical classification system; AChEI = Acetylcholine-esterase inhibitor; OCS = oral corticosteroids; NSIST = non-steroidal immunosuppressant therapy

## Table S2. Medication Use Periods and Definitions

| **Short code** | **Explanation** | **Definition** | **Length** |
| --- | --- | --- | --- |
| PRE12 | 9-12 months before preg. | lmp-365 to lmp-274 | 92 days |
| PRE9 | 6-9 months before preg. | lmp-273 to lmp-183 | 91 days |
| PRE6 | 3-6 months before preg. | lmp-182 to lmp-92 | 91 days |
| PRE3 | 0-3 months before preg. | lmp-91 to lmp-1 | 91 days |
| t1 | first trimester | lmp to lmp+90 | 91 days |
| t2 | second trimester | lmp+91 to lmp+195 | 105 days for term births (min. 64 days corresponding to birth at 22 gestational weeks) |
| t3 | third trimester | lmp+196 to deliv_date-1 | 86-96 days for term pregnancies (corresponding to gestational age 40+3 to 42+0) |
| POST1 | 0-3 months postpartum | deliv_date to deliv_date+90 | 91 days |
| POST2 | 3-6 months postpartum | deliv_date+91 to deliv_date+182 | 91 days |
| **Aggregated periods:** | | |  |
| base | Baseline/pre-pregnancy: 1 year before pregnancy | lmp-365 to lmp-1 | 364 days |
| preg | during pregnancy | lmp to deliv_date-1  (lmp to deliv_date for hospital contacts) | 154-308 days (corresponding to 22-44 weeks) |
| post | 6 months postpartum | deliv_date to deliv_date+182  (deliv_date+1 to deliv_date+182 for hospital contacts) | 182 days |

Abbreviations: lmp = last menstrual period, deliv_date = delivery date

## Table S3. Autoimmune Diseases Included in the Autoimmune Comorbidity Score, based on Fang et.al 2015 (doi:10.1111/joim.12310)

| **Condition** | **ICD-10 Code** | **Description from ICD-10 Version: 2019** |
| --- | --- | --- |
| Polymyositis/Dermatomyositis | M33 | Juvenile dermatomyositis (M33.0), Other dermatomyositis (M33.1), Polymyositis (M33.2), Dermatopolymyositis, unspecified (M33.9) |
| Systemic lupus erythematosus | M32, **except M32.0** | M32.0: Drug-induced systemic lupus erythematosus |
|  |  | M32.1: Systemic lupus erythematosus with organ or system involvement |
|  |  | M32.8: Other forms of systemic lupus erythematosus |
|  |  | M32.9: Systemic lupus erythematosus, unspecified |
| Addison’s disease | E27.1 | E27.1 Primary adrenocortical insufficiency (Addison’s) |
|  |  | E27.2: Addisonian crisis/ Adrenal crisis |
|  |  | E27.3: Drug-induced adrenocortical insufficiency |
|  |  | E27.4: Other and unspecified adrenocortical insufficiency |
|  |  | E27.5: Adrenomedullary hyperfunction |
|  |  | E27.8: Other specified disorders of adrenal gland |
|  |  | E27.9: Disorder of adrenal gland, unspecified |
| Guillain-Barre syndrome | G61.0 | Guillain-Barre syndrome |
| Juvenile rheumatoid arthritis (Edit Jenny: including all juvenile arthritis) | M08, M09 | M08 - Juvenile arthritis |
|  |  | M08.0: Juvenile rheumatoid arthritis |
|  |  | M08.1: Juvenile ankylosing spondylitis |
|  |  | M08.2: Juvenile arthritis with systemic onset |
|  |  | M08.3: Juvenile polyarthritis (seronegative) |
|  |  | Chronic juvenile polyarthritis |
|  |  | M08.4: Pauciarticular juvenile arthritis |
|  |  | M08.8: Other juvenile arthritis |
|  |  | M08.9: Juvenile arthritis, unspecified |
|  |  |  |
|  |  | M09 - Juvenile arthritis in diseases classified elsewhere |
|  |  | M09.0*: Juvenile arthritis in psoriasis (L40.5†) |
|  |  | M09.1*: Juvenile arthritis in Crohn disease [regional enteritis] (K50.-†) |
|  |  | M09.2*: Juvenile arthritis in ulcerative colitis (K51.-†) |
|  |  | M09.8*: Juvenile arthritis in other diseases classified elsewhere |
| Multiple sclerosis | G35 | Multiple sclerosis |
| Vitiligo | L80 | Vitiligo |
| Idiopathic thrombocytopenic purpura | D69.3 | Idiopathic thrombocytopenic purpura/ Evans syndrome |
| Reiter’s syndrome (reactive arthritis) | M02.3 | Reiter's disease |
| Pernicious anaemia | D51.0 | Vitamin B12 deficiency anemia due to intrinsic factor deficiency |
| Hypothyroidism | E03.9 | Hypothyroidism, unspecified |
| Hyperthyroidism | E05 | E05 - Thyrotoxicosis [hyperthyroidism] |
|  |  | E05.0: Thyrotoxicosis with diffuse goitre |
|  |  | Exophthalmic or toxic goitre NOS |
|  |  | Graves disease |
|  |  | Toxic diffuse goitre: E05.1 |
|  |  | Thyrotoxicosis with toxic single thyroid nodule |
|  |  | Thyrotoxicosis with toxic uninodular goitre |
|  |  | E05.2: Thyrotoxicosis with toxic multinodular goitre |
|  |  | Toxic nodular goitre NOS: E05.3 |
|  |  | Thyrotoxicosis from ectopic thyroid tissue |
|  |  | E05.4: Thyrotoxicosis factitia |
|  |  | E05.5: Thyroid crisis or storm |
|  |  | E05.8: Other thyrotoxicosis |
|  |  | E05.9: Thyrotoxicosis, unspecified |
| Polymyalgia rheumatica | M35.3, M31.5 | M35.3: Polymyalgia rheumatica |
|  |  | M31.5: Giant cell arteritis with polymyalgia rheumatica |
| Biliary cirrhosis | K74.3 | Primary biliary cirrhosis |
| Raynaud | I73.0 | Raynaud's syndrome |
| Temporal arteritis | M31.6 | Other giant cell arteritis |
| Ankylosing spondylitis | M45 | Ankylosing spondylitis |
| Adult rheumatoid arthritis | M05, M06 | M05 - Seropositive rheumatoid arthritis |
|  |  | M05.1:Rheumatoid lung disease (J99.0*) |
|  |  | M05.2:Rheumatoid vasculitis |
|  |  | M05.3:Rheumatoid arthritis with involvement of other organs and systems |
|  |  | M05.8:Other seropositive rheumatoid arthritis |
|  |  | M05.9:Seropositive rheumatoid arthritis, unspecified |
|  |  |  |
|  |  | M06 - Other rheumatoid arthritis |
|  |  | M06.0:Seronegative rheumatoid arthritis |
|  |  | M06.1:Adult-onset Still disease |
|  |  | M06.2:Rheumatoid bursitis |
|  |  | M06.3:Rheumatoid nodule |
|  |  | M06.4:Inflammatory polyarthropathy |
|  |  | M06.8Other specified rheumatoid arthritis |
|  |  | M06.9:Rheumatoid arthritis, unspecified |
| Sjøgren’s syndrome | M35.0 | Sicca syndrome [Sjögren] |
| Psoriasis | L40 | L40 - Psoriasis |
|  |  | L40.0: Psoriasis vulgaris/Nummular psoriasis/Plaque psoriasis |
|  |  | L40.1:Generalized pustular psoriasis/Impetigo herpetiformis/Von Zumbusch disease |
|  |  | L40.2:Acrodermatitis continua |
|  |  | L40.3:Pustulosis palmaris et plantaris |
|  |  | L40.4:Guttate psoriasis |
|  |  | L40.5†:Arthropathic psoriasis (M07.0-M07.3*, M09.0*) |
|  |  | L40.8:Other psoriasis, Flexural psoriasis |
|  |  | L40.9: Psoriasis, unspecified |
| Autoimmune thyroiditis | E06.3 | Autoimmune thyroiditis |
| Type I Diabetes mellitus | E10 | Type 1 diabetes mellitus |
| Crohn’s disease | K50 | Crohn's disease |
| Ulcerative colitis | K51 | Ulcerative colitis |
| Sarcoidosis | D86 | Sarcoidosis |
| Coeliac disease | K90.0 | Coeliac disease |

## Table S4. Code to Set Up and Run the Trajectory Model in Stata

Stata code:

```

*generate time-intervals for gbtm:

gen int1=-9 //9-12 months before
gen int2=-6 //6-9 months before

gen int3=-3 //6-3 months before

gen int4=0 //0-3 months before

gen int5=3 //t1

gen int6=6 //t2

gen int7=9 //t3

*run the model

traj if user==1, multgroups(4) ///

var1(PRE12_SYMPT PRE9_SYMPT PRE6_SYMPT PRE3_SYMPT T1_SYMPT T2_SYMPT T3_SYMPT) indep1(int*) model1(logit) order(2 1 1 1) ///

var2(PRE12_OCS PRE9_OCS PRE6_OCS PRE3_OCS T1_OCS T2_OCS T3_OCS) indep2(int*) model2(logit) order(1 1 1 1) ///

var3(PRE12_NSIST PRE9_NSIST PRE6_NSIST PRE3_NSIST T1_NSIST T2_NSIST T3_NSIST) indep3(int*) model3(logit) order(1 1 2 2)

*plot the model

multtrajplot, ytitle1(“Sympt. med.”) ytitle2(“OCS”) ytitle3(“NSIST”) xlabel(-9 -6 -3 0 3 6 9) xtitle (“months relative to LMP”) ci

```

## Table S5. Examples of Alternative Models and Model Fit Statistics

| Number of groups | 5 | 4 | 4 | 4 | 3 |
| --- | --- | --- | --- | --- | --- |
| Polynomial settings | SYMPT(1 1 1 1 1) OCS(1 1 1 1 1) NSIST(1 1 1 1 1) | SYMPT( 1 1 1 1)  OCS( 1 1 1 1)  NSIST( 1 1 1 1) | SYMPT( 2 1 1 1)  OCS( 1 1 1 1)  NSIST( 1 1 2 2) | SYMPT( 1 1 1 1)  OCS( 1 1 1 1)  NSIST( 1 1 3 3) | SYMPT( 1 1 1)  OCS( 1 1 1)  NSIST( 1 1 1) |
| BIC | -1,718.7 | -1,746.8 | -1,754.3 | -1,757.8 | -1,781.2 |
| Group 1 Size | 15.2% | 35.8% | 35.8% | 35.9% | 43.9% |
| OCC Group 1 | 104.1 | 26.6 | 38.2 | 38.4 | 31.6 |
| Group 2 Size | 31.4% | 32.2% | 32.4% | 32.1% | 37.6% |
| OCC Group 2 | 47.2 | 24.7 | 37.5 | 34.1 | 72.9 |
| Group 3 Size | 32.2% | 17.7% | 17.8% | 17.8% | 18.5% |
| OCC Group 3 | 34.3 | 728.4 | 1067.1 | 1027.4 | 717.1 |
| Group 4 Size | 15.2% | 14.3% | 14.1% | 14.2% | - |
| OCC Group 4 | 189.4 | 127.1 | 141.5 | 160.1 | - |
| Group 5 Size | 6.0% | - | - | - | - |
| OCC Group 5 | 9993.4 | - | - | - | - |

Footnotes:
BIC = Bayesian information criterion (for data, i.e. for number of data points, not number of cases), the lower the “better”; OCC = The odds of correct classification.
Abbreviations: SYMPT = Symptomatic medication; OCS = oral corticosteroid; NSIST = non-steroidal immunosuppressant therapy

## Table S6. Clinical and Demographic Background Characteristics for the MG Pregnancy Cohort, Pooled and By Country

| **Characteristic** | **All** | **Norway** | **Sweden** | **Standardized difference^a^** |
| --- | --- | --- | --- | --- |
| All | (n=339) | (n=108) | (n=231) |  |
| % | 100.0 % | 31.9 % | 68.1 % | -0.74 |
| Mother's age at the time of delivery (years): |  |  |  |  |
| -19-30 | 131 (38.6%) | 38 (35.2%) | 93 (40.3%) | -0.11 |
| -31-34 | 112 (33.0%) | 38 (35.2%) | 74 (32.0%) | 0.07 |
| -35-43 | 96 (28.3%) | 32 (29.6%) | 64 (27.7%) | 0.04 |
| Mother's age, Median (IQR) | 32 (28-35) | 32 (29-35) | 32 (28-35) |  |
| Mother's age, Mean±SD | 31.7±4.7 | 31.8±4.3 | 31.7±4.8 | 0.02 |
| Year of delivery: (Range) | 2007 - 2023 | 2010 - 2023 | 2007 - 2022 |  |
| -2007-2013 | 121 (35.7%) | 29 (26.9%) | 92 (39.8%) | -0.27 |
| -2014-2018 | 114 (33.6%) | 34 (31.5%) | 80 (34.6%) | -0.07 |
| - 2019-2023 | 104 (30.7%) | 45 (41.7%) | 59 (25.5%) | 0.35 |
| Source Country: |  |  |  |  |
| -Norway | 108 (31.9%) | 108 (100.0%) | - |  |
| -Sweden | 231 (68.1%) | - | 231 (100.0%) |  |
| Number of previous births: |  |  |  |  |
| -0 | 151 (44.5%) | 48 (44.4%) | 103 (44.6%) | 0.00 |
| -1 | 138 (40.7%) | 42 (38.9%) | 96 (41.6%) | -0.06 |
| -2 or more | 50 (14.7%) | 18 (16.7%) | 32 (13.9%) | 0.08 |
| Previous stillbirth(s) | 5 (1.5%) | <5 | <5 | 0.13 |
| Mother born outside the Nordics | 83 (24.5%) | 24 (22.2%) | 59 (25.5%) | -0.08 |
| Mother cohabiting | 308 (90.9%) | 102 (94.4%) | 206 (89.2%) | 0.19 |
| Mother's education level: |  |  |  |  |
| -Bachelor, Master, or higher | 150 (44.2%) | 72 (66.7%) | 78 (33.8%) | 0.67 |
| -Secondary | 144 (42.5%) | 21 (19.4%) | 123 (53.2%) | -0.72 |
| -Compulsory or less | 36 (10.6%) | 13 (12.0%) | 23 (10.0%) | 0.06 |
| -Missing | 9 (2.7%) | <5 | 7 (3.0%) | 0.04 |
| Mother's BMI at first contact during pregnancy, (Mean±SD) | 25.3±5.2 | 25.2±6.1 | 25.3±4.8 | -0.02 |
| BMI is missing | 36 (10.6%) | 25 (23.1%) | 11 (4.8%) | 0.56 |
| Smoking at the start of pregnancy: |  |  |  |  |
| -Yes | - | <5 | 10 (4.3%) | -0.03 |
| -No | 297 (87.6%) | 86 (79.6%) | 211 (91.3%) | -0.34 |
| -Missing | - | - | 10 (4.3%) | #I/T |
| Smoking at the end of pregnancy: |  |  |  |  |
| -Yes | - | <5 | <5 | 0.13 |
| -No | 277 (81.7%) | 83 (76.9%) | 194 (84.0%) | -0.18 |
| -Missing | - | - | - |  |
| Folic acid supplementation before pregnancy (only for Norway) | - | 49 (45.4%) | - | #I/T |
| Assisted Reproductive Technology | 23 (6.8%) | 10 (9.3%) | 13 (5.6%) | 0.14 |
| Preterm birth (<37 gw) | 30 (8.8%) | 12 (11.1%) | 18 (7.8%) | 0.11 |
| Maternal comorbities: |  |  |  |  |
| -Chronic asthma | 29 (8.6%) | 7 (6.5%) | 22 (9.5%) | -0.11 |
| -Pre-existing hypertension | <5 | 0 (0.0%) | <5 | -0.26 |
| -Chronic renal disease | <5 | <5 | 0 (0.0%) | 0.39 |
| -Epilepsy | <5 | 0 (0.0%) | <5 | -0.26 |
| -Diabetes | 5 (1.5%) | <5 | <5 | 0.13 |
| -Gestational hypertension | 8 (2.4%) | <5 | <5 | 0.13 |
| -Autoimmune comorbidity^c^ | 58 (17.1%) | 23 (21.3%) | 35 (15.2%) | 0.16 |
| Autoimmune comorbidity score, Range | 0 - 5 | 0 - 3 | 0 - 5 |  |
| Polypharmacy count, ^b^ (Mean±SD) | 6.3±4.6 | 6.4±5.0 | 6.2±4.4 | 0.04 |
| Overall medication use by ATC-classes (Mean±SD): ^d^ |  |  |  |  |
| A Alimentary Tract And Metabolism | 0.4±0.5 | 0.5±0.5 | 0.4±0.5 | 0.04 |
| B Blood And Blood Forming Organs | 0.3±0.4 | 0.3±0.4 | 0.3±0.4 | 0.20 |
| C Cardiovascular System | 0.1±0.3 | 0.1±0.3 | 0.1±0.3 | 0.00 |
| D Dermatologicals | 0.2±0.4 | 0.2±0.4 | 0.2±0.4 | 0.00 |
| G Genito Urinary System And Sex Hormones | 0.4±0.5 | 0.5±0.5 | 0.4±0.5 | 0.00 |
| H Systemic Hormonal Preparations, Excl. Sex Hormones And Insulins | 0.4±0.5 | 0.4±0.5 | 0.4±0.5 | 0.20 |
| J Anti-infectives For Systemic Use | 0.5±0.5 | 0.5±0.5 | 0.5±0.5 | 0.00 |
| L Antineoplastic And Immunomodulating Agents | 0.2±0.4 | 0.3±0.4 | 0.2±0.4 | 0.00 |
| M Musculo-Skeletal System | 0.2±0.4 | 0.2±0.4 | 0.2±0.4 | 0.25 |
| N Nervous System | 0.7±0.5 | 0.6±0.5 | 0.7±0.5 | 0.00 |
| P Antiparasitic Products, Insecticides And Repellents | 0.0±0.2 | 0.1±0.3 | 0.0±0.2 | -0.20 |
| R Respiratory System | 0.5±0.5 | 0.5±0.5 | 0.5±0.5 | 0.50 |
| S Sensory Organs | 0.2±0.4 | 0.2±0.4 | 0.2±0.4 | 0.00 |
| V Various | 0.0±0.1 | 0.0±0.2 | 0.0±0.1 | 0.00 |

Footnotes:

^a^ Cohen's h for proportions ( $\boldsymbol{h}=\boldsymbol{2}\boldsymbol{arcsin}(\sqrt{\boldsymbol{p}_{\boldsymbol{Norway}}}- \boldsymbol{2}\boldsymbol{arcsin}(\sqrt{\boldsymbol{p}_{\boldsymbol{Sweden}}}$ ) and Cohen’s d for means ( $\boldsymbol{d}= \frac{(\boldsymbol{Mean}_{\boldsymbol{Norway}}- \boldsymbol{Mean}_{\boldsymbol{Sweden}})}{\boldsymbol{SD}_{\boldsymbol{total}}}$ ).

^b^ The polypharmacy count was defined as the number of distinct medications during pre-pregnancy or pregnancy at the 3rd ATC-level (pharmacological subgroup), including both MG and non-MG medications.

^c^ Autoimmune comorbidity was defined from NPR as at least one autoimmune condition ever recorded before LMP among 26 predefined autoimmune conditions known to be associated with MG (Table S3), and the autoimmune comorbidity score was defined as the number of distinct conditions.

^d^ The number of distinct medications at the 3rd ATC-level, stratified by the 1st ATC-level (anatomical main group).

Abbreviations: n = number; SD = standard deviation; BMI = body mass index; gw = gestational weeks; ART = assisted reproductive technology; ATC = Anatomical Therapeutic Chemical classification system; NPR = National Patient Registry; LMP = last menstrual period.

## Table S7. Proportion Medication Users of Each Medication and Medication Class in Each Drug Use Period, Pooled and By Country

| Proportion users of each medication/medication group in each drug use period among all pregnancies in cohort - Pooled. | | | | | | | | | | | | | | |
| --- | --- | --- | --- | --- | --- | --- | --- | --- | --- | --- | --- | --- | --- | --- |
|  | PRE12 | PRE9 | PRE6 | PRE3 | t1 | t2 | t3 | POST1 | POST2 | base | preg | post | ever |  |
| ANY | 37 % | 36 % | 37 % | 38 % | 33 % | 35 % | 34 % | 32 % | 36 % | 55 % | 46 % | 43 % | 63 % |  |
| SYMPT | 29 % | 29 % | 31 % | 29 % | 25 % | 27 % | 26 % | 25 % | 29 % | 46 % | 36 % | 34 % | 53 % |  |
| MEST | 28 % | 29 % | 30 % | 27 % | 24 % | 26 % | 24 % | 25 % | 28 % | 44 % | 35 % | 33 % | 51 % |  |
| MYT | 1 % | <1% | <1% | 1 % | <1% | <1% | <1% | <1% | <1% | 2 % | 1 % | <1% | 3 % |  |
| EPH | <1% | <1% | <1% | <1% | <1% | 0 % | 0 % | 0 % | 0 % | 2 % | <1% | 0 % | 2 % |  |
| PRED | 10 % | 9 % | 7 % | 7 % | 9 % | 8 % | 8 % | 9 % | 12 % | 18 % | 13 % | 15 % | 24 % |  |
| NSIST | 8 % | 9 % | 9 % | 9 % | 8 % | 5 % | 4 % | 6 % | 9 % | 13 % | 10 % | 12 % | 16 % |  |
| AZA | 6 % | 8 % | 7 % | 8 % | 7 % | 5 % | 4 % | 5 % | 7 % | 11 % | 9 % | 9 % | 13 % |  |
| CSPA | <1% | <1% | <1% | <1% | <1% | <1% | <1% | <1% | 1 % | <1% | <1% | 2 % | 2 % |  |
| TAC | <1% | <1% | <1% | <1% | 0 % | 0 % | 0 % | 0 % | <1% | <1% | 0 % | <1% | <1% |  |
| MMF | <1% | 0 % | 0 % | 0 % | 0 % | 0 % | 0 % | 0 % | 0 % | <1% | 0 % | 0 % | <1% |  |
| MTX | <1% | 0 % | 0 % | 0 % | 0 % | 0 % | 0 % | 0 % | 0 % | <1% | 0 % | 0 % | <1% |  |
| Proportion users of each medication/medication group in each drug use period among all pregnancies in Norway | | | | | | | | | | | | | | |
|  | PRE12 | PRE9 | PRE6 | PRE3 | t1 | t2 | t3 | POST1 | POST2 | base | preg | post | ever |  |
| ANY | 38 % | 33 % | 32 % | 39 % | 34 % | 32 % | 30 % | 31 % | 35 % | 53 % | 47 % | 42 % | 61 % |  |
| SYMPT | 26 % | 25 % | 24 % | 24 % | 21 % | 20 % | 22 % | 21 % | 28 % | 40 % | 32 % | 31 % | 47 % |  |
| MEST | 24 % | 25 % | 24 % | 23 % | 21 % | 20 % | 22 % | 21 % | 28 % | 37 % | 32 % | 31 % | 44 % |  |
| MYT | <5% | 0 % | 0 % | 0 % | 0 % | 0 % | 0 % | 0 % | 0 % | <5% | 0 % | 0 % | <5% |  |
| EPH | <5% | 0 % | 0 % | <5% | 0 % | 0 % | 0 % | 0 % | 0 % | <5% | 0 % | 0 % | <5% |  |
| PRED | 16 % | 9 % | 11 % | 9 % | 12 % | 6 % | 6 % | 9 % | 17 % | 26 % | 15 % | 19 % | 34 % |  |
| NSIST | 15 % | 15 % | 15 % | 18 % | 17 % | 12 % | 7 % | 14 % | 15 % | 22 % | 20 % | 20 % | 25 % |  |
| AZA | 14 % | 15 % | 15 % | 18 % | 17 % | 12 % | 7 % | 14 % | 15 % | 21 % | 20 % | 20 % | 24 % |  |
| CSPA | 0 % | 0 % | 0 % | 0 % | 0 % | 0 % | 0 % | 0 % | 0 % | 0 % | 0 % | 0 % | 0 % |  |
| TAC | 0 % | 0 % | 0 % | 0 % | 0 % | 0 % | 0 % | 0 % | 0 % | 0 % | 0 % | 0 % | 0 % |  |
| MMF | <5% | 0 % | 0 % | 0 % | 0 % | 0 % | 0 % | 0 % | 0 % | <5% | 0 % | 0 % | <5% |  |
| MTX | 0 % | 0 % | 0 % | 0 % | 0 % | 0 % | 0 % | 0 % | 0 % | 0 % | 0 % | 0 % | 0 % |  |
| Proportion users of each medication/medication group in each drug use period among all pregnancies in Sweden | | | | | | | | | | | | | | |
|  | PRE12 | PRE9 | PRE6 | PRE3 | t1 | t2 | t3 | POST1 | POST2 | base | preg | post | ever |  |
| ANY | 37 % | 38 % | 40 % | 37 % | 33 % | 36 % | 35 % | 33 % | 36 % | 56 % | 46 % | 44 % | 63 % |  |
| SYMPT | 31 % | 32 % | 34 % | 31 % | 27 % | 30 % | 27 % | 27 % | 29 % | 48 % | 37 % | 35 % | 55 % |  |
| MEST | 30 % | 31 % | 33 % | 29 % | 26 % | 29 % | 26 % | 27 % | 28 % | 47 % | 35 % | 34 % | 54 % |  |
| MYT | <2% | <2% | <2% | 2 % | <2% | <2% | <2% | <2% | <2% | 3 % | 2 % | <2% | 4 % |  |
| EPH | <2% | <2% | <2% | <2% | <2% | 0 % | 0 % | 0 % | 0 % | <2% | <2% | 0 % | <2% |  |
| PRED | 8 % | 10 % | 6 % | 6 % | 8 % | 9 % | 9 % | 8 % | 10 % | 14 % | 12 % | 13 % | 19 % |  |
| NSIST | 5 % | 6 % | 6 % | 5 % | 3 % | <2% | 3 % | 3 % | 6 % | 9 % | 5 % | 8 % | 12 % |  |
| AZA | 2 % | 4 % | 4 % | 3 % | 3 % | <2% | 2 % | <2% | 4 % | 6 % | 3 % | 4 % | 8 % |  |
| CSPA | <2% | <2% | <2% | <2% | <2% | <2% | <2% | <2% | 2 % | <2% | <2% | 3 % | 3 % |  |
| TAC | <2% | <2% | <2% | <2% | 0 % | 0 % | 0 % | 0 % | <2% | <2% | 0 % | <2% | <2% |  |
| MMF | 0 % | 0 % | 0 % | 0 % | 0 % | 0 % | 0 % | 0 % | 0 % | 0 % | 0 % | 0 % | 0 % |  |
| MTX | <2% | 0 % | 0 % | 0 % | 0 % | 0 % | 0 % | 0 % | 0 % | <2% | 0 % | 0 % | <2% |  |

## Table S8. Clinical and Demographic Background Characteristics for Cohort, and Separately by Pregnancies with Any MG Medication Use During Pre-pregnancy and Pregnancy (‘Users’) vs. No MG Medication Use (‘Non-users’)

| Characteristic | **All** | **‘Users’** | **‘Non-users’** | **Standardized difference^a^** |
| --- | --- | --- | --- | --- |
| All | (n=339) | (n=201) | (n=138) |  |
| % | 100.0 % | 59.3 % | 40.7 % |  |
| Mother's age at the time of delivery (years): |  |  |  |  |
| -19-30 | 131 (38.6%) | 81 (40.3%) | 50 (36.2%) | 0.08 |
| -31-34 | 112 (33.0%) | 63 (31.3%) | 49 (35.5%) | -0.09 |
| -35-43 | 96 (28.3%) | 57 (28.4%) | 39 (28.3%) | 0.00 |
| Mother's age, Median (IQR) | 32 (28-35) | 32 (28-35) | 32 (29-35) |  |
| Mother's age, (Mean±SD) | 31.7±4.7 | 31.8±4.6 | 31.6±4.8 | 0.04 |
| Year of delivery: (Range) | 2007 - 2023 | 2007 - 2023 | 2007 - 2023 |  |
| -2007-2013 | 121 (35.7%) | 76 (37.8%) | 45 (32.6%) | 0.11 |
| -2014-2018 | 114 (33.6%) | 64 (31.8%) | 50 (36.2%) | -0.09 |
| -2019-2023 | 104 (30.7%) | 61 (30.3%) | 43 (31.2%) | -0.02 |
| Source Country: |  |  |  |  |
| -Norway | 108 (31.9%) | 64 (31.8%) | 44 (31.9%) | 0.00 |
| -Sweden | 231 (68.1%) | 137 (68.2%) | 94 (68.1%) | 0.00 |
| Number of previous births: |  |  |  |  |
| -0 | 151 (44.5%) | 96 (47.8%) | 55 (39.9%) | 0.16 |
| -1 | 138 (40.7%) | 77 (38.3%) | 61 (44.2%) | -0.12 |
| -2 or more | 50 (14.7%) | 28 (13.9%) | 22 (15.9%) | -0.06 |
| Previous stillbirth(s) | 5 (1.5%) | <5 | <5 | -0.06 |
| Mother born outside the Nordics | 83 (24.5%) | 58 (28.9%) | 25 (18.1%) | 0.26 |
| Mother cohabiting | 308 (90.9%) | 179 (89.1%) | 129 (93.5%) | -0.16 |
| Mother's education level: |  |  |  |  |
| -Bachelor, Master, or higher | 150 (44.2%) | 76 (37.8%) | 74 (53.6%) | -0.32 |
| - Secondary | 144 (42.5%) | 92 (45.8%) | 52 (37.7%) | 0.16 |
| -Compulsory or less | 36 (10.6%) | 25 (12.4%) | 11 (8.0%) | 0.15 |
| - Missing | 9 (2.7%) | 8 (4.0%) | <5 | 0.06 |
| Mother's BMI at first contact during pregnancy, Mean±SD | 25.3±5.2 | 25.4±5.1 | 25.0±5.4 | 0.08 |
| BMI is missing | 36 (10.6%) | 20 (10.0%) | 16 (11.6%) | -0.05 |
| Smoking at the start of pregnancy: |  |  |  |  |
| -Yes | - | 10 (5.0%) | <5 | 0.11 |
| - No | 297 (87.6%) | 168 (83.6%) | 129 (93.5%) | -0.32 |
| -Missing | - | 23 (11.4%) | - | #I/T |
| Smoking at the end of pregnancy: |  |  |  |  |
| -Yes | - | 6 (3.0%) | <5 | 0.01 |
| -No | 277 (81.7%) | 161 (80.1%) | 116 (84.1%) | -0.10 |
| -Missing | - | 34 (16.9%) | - | #I/T |
| Folic acid supplementation before pregnancy (only for Norway) | 49 (14.5%) | 25 (12.4%) | 24 (17.4%) | -0.20 |
| Assisted Reproductive Technology | 23 (6.8%) | 16 (8.0%) | 7 (5.1%) | 0.12 |
| Preterm birth (<37 gw) | 30 (8.8%) | 20 (10.0%) | 10 (7.2%) | 0.10 |
| Maternal comorbities: |  |  |  |  |
| -Chronic asthma | 29 (8.6%) | 22 (10.9%) | 7 (5.1%) | 0.22 |
| -Pre-existing hypertension | <5 | <5 | <5 | -0.06 |
| -Chronic renal disease | <5 | <5 | 0 (0.0%) | 0.28 |
| -Epilepsy | <5 | <5 | <5 | -0.06 |
| -Diabetes | 5 (1.5%) | <5 | <5 | -0.06 |
| -Gestational hypertension | 8 (2.4%) | 5 (2.5%) | <5 | -0.02 |
| -Autoimmune comorbidity (yes/no)^c^ | 58 (17.1%) | 34 (16.9%) | 24 (17.4%) | -0.01 |
| Autoimmune comorbidity score, Range | 0 - 5 | 0 - 5 | 0 - 3 |  |
| Polypharmacy count ^b^ (Mean±SD) | 6.3±4.6 | 7.8±4.7 | 4.0±3.5 | 0.83 |
| Overall medication use by ATC-classes (Mean±SD): ^d^ |  |  |  |  |
| A Alimentary Tract And Metabolism | 0.4±0.5 | 0.6±0.5 | 0.2±0.4 | 0.80 |
| B Blood And Blood Forming Organs | 0.3±0.4 | 0.3±0.5 | 0.2±0.4 | 0.25 |
| C Cardiovascular System | 0.1±0.3 | 0.1±0.3 | 0.1±0.3 | 0.00 |
| D Dermatologicals | 0.2±0.4 | 0.2±0.4 | 0.1±0.4 | 0.25 |
| G Genito Urinary System And Sex Hormones | 0.4±0.5 | 0.4±0.5 | 0.4±0.5 | 0.00 |
| H Systemic Hormonal Preparations, Excl. Sex Hormones And Insulins | 0.4±0.5 | 0.5±0.5 | 0.2±0.4 | 0.60 |
| J Antiinfectives For Systemic Use | 0.5±0.5 | 0.6±0.5 | 0.4±0.5 | 0.40 |
| L Antineoplastic And Immunomodulating Agents | 0.2±0.4 | 0.3±0.4 | 0.1±0.3 | 0.50 |
| M Musculo-Skeletal System | 0.2±0.4 | 0.2±0.4 | 0.2±0.4 | 0.00 |
| N Nervous System | 0.7±0.5 | 0.9±0.3 | 0.3±0.5 | 1.20 |
| P Antiparasitic Products, Insecticides And Repellents | 0.0±0.2 | 0.0±0.2 | 0.0±0.2 | 0.00 |
| R Respiratory System | 0.5±0.5 | 0.5±0.5 | 0.4±0.5 | 0.20 |
| S Sensory Organs | 0.2±0.4 | 0.2±0.4 | 0.2±0.4 | 0.00 |
| V Various | 0.0±0.1 | 0.0±0.1 | 0.0±0.1 | 0.00 |

Footnotes:

^a^ Cohen's h for proportions ( $\boldsymbol{h}=\boldsymbol{2}\boldsymbol{arcsin}(\sqrt{\boldsymbol{p}_{User}}- \boldsymbol{2arcsin}(\sqrt{\boldsymbol{p}_{\boldsymbol{Non-user}}}$ ) and Cohen’s d for means ( $\boldsymbol{d}= \frac{(\boldsymbol{Mean}_{\boldsymbol{User}}- \boldsymbol{Mean}_{\boldsymbol{Non-user}})}{\boldsymbol{SD}_{\boldsymbol{total}}}$ ).

^b^ The polypharmacy count was defined as the number of distinct medications during pre-pregnancy or pregnancy at the 3rd ATC-level (pharmacological subgroup), including both MG and non-MG medications.

^c^ Autoimmune comorbidity was defined from NPR as at least one autoimmune condition ever recorded before LMP among 26 predefined autoimmune conditions known to be associated with MG (Table S3), and the autoimmune comorbidity score was defined as the number of distinct conditions.

^d^ The number of distinct medications at the 3rd ATC-level, stratified by the 1st ATC-level (anatomical main group).

Abbreviations: n = number; SD = standard deviation; BMI = body mass index; gw = gestational weeks; ART = assisted reproductive technology; ATC = Anatomical Therapeutic Classification; NPR = National Patient Registry; LMP = last menstrual period.

## Table S9. Polynomial Settings, Model Fit Statistics, and GBMTM Results for the Final Main Model

| **Polynomial terms used:** | SYMPT(3 1 1 1)  OCS(3 1 1 1)  NSIST(1 1 3 3) | | | |
| --- | --- | --- | --- | --- |
| **BIC for the whole model (N=4221):** | -1769.4 | | | |
|  | **Group 1** | **Group 2** | **Group 3** | **Group 4** |
| **Group membership (%)** | 35.8 | 32.3 | 17.8 | 14.1 |
| **APP** | 0.956 | 0.948 | 0.997 | 0.963 |
| **Min. PP** | 0.620 | 0.603 | 0.965 | 0.665 |
| **Max. PP** | 1.000 | 1.000 | 1.000 | 1.000 |
| **OCC** | 40.0 | 36.4 | 1695.3 | 158.6 |

Footnotes:
Model included all ‘Users’ (N=201), while ‘Non-users’ (N=138) were kept out of sample.
Polynomial terms: 1 = 1st exponential function (line), 2 = quadratic function (parabol), 3 = qubic function (S-shape).

Abbreviations:

GBMTM = Group Based Multi-Trajectory Modelling; SYMPT = Symptomatic medication; OCS = oral corticosteroid; NSIST = non-steroidal immunosuppressant therapy; BIC = Bayesian information criterion (for number of data points, not number of cases. The lower the “better”); APP=Average posterior probability (i.e. the mean probability to be assigned group X among all those who were assigned group X, the higher the “better”); Min.PP/ Max.PP = minimum and maximum posterior probability to be assigned the group that they were actually assigned (the higher the “better”); OCC = The odds of correct classification (i.e. the odds of being assigned the group based on posterior probability, divided by the odds of being in the group, computed by group as (APP/(1-APP))/(n_group /(N – n_group)), the higher the “better”, where N=201).

## Table S10. Polynomial Settings, Model Fit Statistics, and GBMTM Results for the Restricted Cohort, i.e. MG Defined Until LMP

| **Polynomial terms used:** | SYMPT(3 1 1 1)  OCS(3 1 1 1)  NSIST(1 1 3 3) | | | |
| --- | --- | --- | --- | --- |
| **BIC for the whole model (N=3906):** | -1663.4 | | | |
|  | **Group 1** | **Group 2** | **Group 3** | **Group 4** |
| **Group membership (%)** | 31.9 | 34.6 | 19.3 | 14.3 |
| **APP** | 0.933 | 0.957 | 0.997 | 0.962 |
| **Min.PP** | 0.576 | 0.519 | 0.957 | 0.658 |
| **Max.PP** | 1.000 | 1.000 | 1.000 | 1.000 |
| **OCC** | 32.5 | 46.2 | 1482.5 | 172.7 |

Footnote:
Model included all ‘Users’ (N=186), while ‘Non-users’ (N=110) were kept out of sample. Polynomial terms: 1 = 1st exponential function (line), 2 = quadratic function (parabol), 3 = qubic function (S-shape);
Abbreviations: GBMTM = Group Based Multi-Trajectory Modelling; MG = Myasthenia Gravis; LMP = last menstrual period; SYMPT = Symptomatic medication; OCS = oral corticosteroid; NSIST = non-steroidal immunosuppressant therapy; BIC = Bayesian information criterion (for number of data points, not number of cases. The lower the “better”); APP=Average posterior probability (i.e. the mean probability to be assigned group X among all those who were assigned group X, the higher the “better”); Min.PP/ Max.PP = minimum and maximum posterior probability to be assigned the group that they were actually assigned (the higher the “better”); OCC = The odds of correct classification (i.e. the odds of being assigned the group based on posterior probability, divided by the odds of being in the group, computed by group as (APP/(1-APP))/(n_group /(N – n_group)), the higher the “better”, where N=186).

Figure S1. Alluvial diagrams illustrating persistence patterns of combination-therapies and monotherapy across pre-pregnancy, pregnancy and postpartum, by trajectory groups.

## Figure S2. Spaghetti plots of individual predicted probabilities.

Figure caption:
Each line represents ≥1 individual's predicted probability of use across all time periods. A wider spread indicates more within-group heterogeneity. E.g. a line close to zero across all time periods indicates very low individual probability of use in all periods (i.e. a persistent non-user of the given medication), while a line close to one across all time periods indicates very high individual probability of use (i.e. a persistent user of the given medication).

STATA code: ´´´

foreach med of local meds {

levelsof _traj_Group, local(trajgroup)

foreach traj of local trajgroup {

melogit `med' i.time if _traj_Group==`traj' || preg_id:

predict p_hat, mu

spagplot p_hat time if _traj_Group==`traj', id(preg_id)

drop p_hat

}

}

´´´

## Figure S3. Mean number of prescription fills of **pyridostigmine** among pyridostigmine users in each drug use period, by trajectory groups.

|  | 'Non-users' | ‘Intermittent users’ | ‘Persistent symptomatic-only users’ | ‘Immuno-suppressant plus symptomatic users’ | ‘Immuno-suppressant-only users’ |
| --- | --- | --- | --- | --- | --- |
| PRE12 | 0 | 1.32 | 2.02 | 2.04 | 1 |
| PRE9 | 0 | 1.25 | 2.24 | 1.83 | 1 |
| PRE6 | 0 | 1.28 | 1.84 | 1.93 | 0 |
| PRE3 | 0 | 1.36 | 1.94 | 1.42 | 0 |
| t1 | 0 | 1.11 | 1.84 | 2.09 | 0 |
| t2 | 0 | 1.89 | 1.71 | 1.89 | 0 |
| t3 | 0 | 1.79 | 1.65 | 1.78 | 0 |
| POST1 | 2.38 | 2.2 | 1.76 | 2.05 | 2 |
| POST2 | 1.33 | 2 | 1.69 | 1.68 | 0 |

## Figure S4. Mean number of prescription fills of prednisolone among prednisolone users in each drug use period, by trajectory groups.

|  | 'Non-users' | ‘Intermittent users’ | ‘Persistent symptomatic-only users’ | ‘Immuno-suppressant plus symptomatic users’ | ‘Immuno-suppressant-only users’ |
| --- | --- | --- | --- | --- | --- |
| PRE12 | 0 | 1.5 | 0 | 1.4 | 1.2 |
| PRE9 | 0 | 1.4 | 1 | 1.86 | 1.17 |
| PRE6 | 0 | 0 | 1 | 1.5 | 1.29 |
| PRE3 | 0 | 1 | 0 | 1.13 | 1.33 |
| t1 | 0 | 1.2 | 0 | 1.36 | 1.4 |
| t2 | 0 | 1.43 | 1 | 1.29 | 1.23 |
| t3 | 0 | 1 | 0 | 1.75 | 1.19 |
| POST1 | 2.33 | 2 | 0 | 1.75 | 1.31 |
| POST2 | 2 | 1.5 | 1 | 1.67 | 1.62 |

## Figure S5. Mean daily dose (mg/day) of prednisolone as the total dispensed dose during period averaged over 91 days among users of prednisolone in each period, by trajectory groups.

|  | 'Non-users' | ‘Intermittent users’ | ‘Persistent symptomatic-only users’ | ‘Immuno-suppressant plus symptomatic users’ | ‘Immuno-suppressant-only users’ |
| --- | --- | --- | --- | --- | --- |
| PRE12 | 0 | 16.26 | 0 | 14.56 | 8.58 |
| PRE9 | 0 | 17.69 | 2.75 | 20.9 | 7.9 |
| PRE6 | 0 | 0 | 0 | 12.77 | 9.42 |
| PRE3 | 0 | 3.02 | 0 | 10.65 | 9.39 |
| t1 | 0 | 14.29 | 0 | 12.76 | 7.42 |
| t2 | 0 | 13.93 | 5.49 | 14.56 | 7.29 |
| t3 | 0 | 8.24 | 0 | 24.04 | 6.81 |
| POST1 | 20.15 | 32.97 | 0 | 28.16 | 9.43 |
| POST2 | 17.4 | 21.31 | 5.63 | 22.69 | 11.53 |

## Figure S6. Mean daily dose (mg/day) of azathioprine as the total dispensed dose during period averaged over 91 days among users of azathioprine in each period, by trajectory groups

|  | 'Non-users' | ‘Intermittent users’ | ‘Persistent symptomatic-only users’ | ‘Immuno-suppressant plus symptomatic users’ | ‘Immuno-suppressant-only users’ |
| --- | --- | --- | --- | --- | --- |
| PRE12 | 0 | 54.95 | 0 | 131.64 | 96.15 |
| PRE9 | 0 | 0 | 0 | 138.13 | 120.19 |
| PRE6 | 0 | 0 | 27.47 | 164.84 | 151.1 |
| PRE3 | 0 | 0 | 0 | 131.35 | 125 |
| t1 | 0 | 54.95 | 0 | 156.59 | 159.68 |
| t2 | 0 | 0 | 0 | 175.52 | 145.21 |
| t3 | 0 | 109.89 | 0 | 182.5 | 126.37 |
| POST1 | 0 | 77.84 | 0 | 149.57 | 135.07 |
| POST2 | 109.89 | 141.94 | 0 | 115.04 | 115.38 |

## Figure S7. Multi-drug trajectory patterns before and during pregnancy, in the restricted birth cohort where myasthenia gravis was recognized before the start of pregnancy.

Figure caption:
Dots describe the actual (observed) proportion of symptomatic MG-medication/ OCS/ NSIST use in each trajectory group. Lines illustrate the predicted probability of use across time periods with 95% confidence intervals. Each ‘User’ pregnancy (N=186) was grouped into one of the four latent groups based on our final GBMTM model. Group sizes were automatically derived from the model, and here shown as the percentage of the whole MG birth cohort (N=296), which included ‘Non-users’ (Group 0, N=110).

Abbreviation: GBMTM = Group-Based Multi-Trajectory Modelling; Pr. = Probability; OCS = oral corticosteroids; NSIST = non-steroidal immunosuppressant therapy; LMP = last menstrual period; T1 = first trimester; T2 = second trimester; T3 = third trimester.
